# Phase controlled synthesis of transition metal carbide nanocrystals by ultrafast flash Joule heating

**DOI:** 10.1038/s41467-021-27878-1

**Published:** 2022-01-11

**Authors:** Bing Deng, Zhe Wang, Weiyin Chen, John Tianci Li, Duy Xuan Luong, Robert A. Carter, Guanhui Gao, Boris I. Yakobson, Yufeng Zhao, James M. Tour

**Affiliations:** 1 Department of Chemistry, Rice University, Houston, TX 77005 USA; 2 Department of Materials Science and NanoEngineering, Rice University, Houston, TX 77005 USA; 3 Smalley-Curl Institute, Rice University, Houston, TX 77005 USA; 4 Corban University, Salem, Oregon 97317 USA; 5 NanoCarbon Center and the Welch Institute for Advanced Materials, Rice University, Houston, TX 77005 USA

**Keywords:** Inorganic chemistry, Electronic materials

## Abstract

Nanoscale carbides enhance ultra-strong ceramics and show activity as high-performance catalysts. Traditional lengthy carburization methods for carbide syntheses usually result in coked surface, large particle size, and uncontrolled phase. Here, a flash Joule heating process is developed for ultrafast synthesis of carbide nanocrystals within 1 s. Various interstitial transition metal carbides (TiC, ZrC, HfC, VC, NbC, TaC, Cr_2_C_3_, MoC, and W_2_C) and covalent carbides (B_4_C and SiC) are produced using low-cost precursors. By controlling pulse voltages, phase-pure molybdenum carbides including β-Mo_2_C and metastable α-MoC_1-x_ and η-MoC_1-x_ are selectively synthesized, demonstrating the excellent phase engineering ability of the flash Joule heating by broadly tunable energy input that can exceed 3000 K coupled with kinetically controlled ultrafast cooling (>10^4^ K s^−1^). Theoretical calculation reveals carbon vacancies as the driving factor for topotactic transition of carbide phases. The phase-dependent hydrogen evolution capability of molybdenum carbides is investigated with β-Mo_2_C showing the best performance.

## Abstract (web summary)

Nanoscale carbides provide access to ultra-strong ceramics and show activity as high-performance catalysts. Here, the authors report a flash Joule heating process for the ultrafast, general synthesis of various transition metal carbides nanocrystals with phase controllability.

## Introduction

Carbides are an important class of materials with broad applications in electronics, ceramics, and energy conversion, due to their extreme hardness, high thermal stability, and widely tunable electronic structures. Nanosized transition metal carbides (TMCs) have been widely used as the precursors for ultra-hard and ultra-strong ceramics^1–3^, high-performance electrochemical catalysts because of their platinum-like electronic structures^4–8^, and catalyst supports due to the strong metal-substrate interactions^9–11^. Traditional methods for bulk carbide syntheses include carburization of metal precursors with gaseous carbon precursors or sintering of metal precursors with graphitic carbon at high temperature^12^. These procedures can be problematic since they result in coked carbide surfaces due to the excessive supply of carbon sources, and large particle sizes with low surface areas that are detrimental to catalytic performance^13,14^.

Much effort has been devoted to synthesizing carbides with fine particle sizes, including temperature-programmed reduction (TPR)^15^, carbothermic reduction of metal precursors^16,17^, laser spray pyrolysis of metal complexes^18^, and solution-based precipitation and carburization^19^. The TPR method is versatile for high-surface-area metal carbide synthesis but requires well-optimized reaction windows^20^. The carbothermic reduction of metal precursors in a furnace is universal in the synthesis of TMCs^16^; however, extended high-temperature conditions are essential to compensate the slow solid-solid reaction kinetics, which inevitably result in sintering or agglomeration^17^. To avoid severe agglomeration, a microwave combustion method is developed for rapid synthesis of Mo_2_C and WC nanodots within 2 min^21^. The pyrolysis of metal complexes involves the use of costly and toxic metal-organic compounds such as Cp_2_Mo_2_(CO)_6_ for the synthesis of Mo_2_C^18,22^, and W(CO)_6_ for the synthesis of WC^23^. The type of carbide is also limited by the availability of volatile metal compounds. The solution-based precipitation and carburization requires long annealing times for full conversion. For example, annealing at 850 °C for 12 to 24 h is needed for the synthesis of MoC using ammonium heptamolybdate ((NH_4_)_6_Mo_7_O_24_·4H_2_O) as the precursor^19^.

Recently, several non-conventional electrical thermal processes have been developed towards energy-efficient high-temperature synthesis^24–26^. The carbothermal shock (CTS) process uses short current pulses for the synthesis of high-entropy alloy nanoparticles on carbon supports at ~2000 K^26^. The ultrahigh temperature sintering (UHS) based on current-induced heating is proposed for sintering and screening of ceramics within 10 s^24^. The spark plasma sintering (SPS) applies an electric current for the reactive carbothermic synthesis of zirconium carbide (ZrC) in 10 min^25^. However, these approaches are targeting the sintering of bulk ceramics and lack the ability in the synthesis of fine nanocrystals. Most importantly, phases and crystal surface structure play significant roles in the behavior of carbides, such as in their hydrogen adsorption/desorption energy^7,27^. However, there are very few procedures to selectively engineer the phases and crystal surfaces of carbides for maximal performance^7,19^.

To meet those demands, here, we develop the ultrafast flash Joule heating (FJH) method for the general synthesis of coke-free and phase-controlled carbide nanocrystals within 1 s. A milliseconds current pulse is passed through the precursors, which brings the sample to an ultrahigh temperature (>3000 K) and then it is rapidly cooled to room temperature (>10^4^ K s^−1^). Thirteen important element carbides are synthesized, including interstitial TMCs of TiC, ZrC, HfC, VC, NbC, TaC, Cr_2_C_3_, MoC, and W_2_C, and covalent carbides of B_4_C and SiC, demonstrating the excellent generality. Moreover, by controlling the FJH pulse voltages, phase-pure molybdenum carbides including thermodynamically stable β-Mo_2_C, and metastable α-MoC_1-x_ and η-MoC_1-x_ are selectively synthesized, showing the phase engineering ability of the synergistic electrical-thermal process. The phase-dependent hydrogen evolution reaction (HER) performance of molybdenum carbides is discovered. The β-Mo_2_C exhibits the best HER performance with an overpotential of –220 mV, Tafel slope of 68 mV dec^−1^, and good durability. This rapid carbide synthesis method could realize low-cost, mass production of nanocrystalline carbides at only 2.2 to 8.6 kJ g^−1^ in electrical energy.

## Results

### Ultrafast synthesis of carbide nanocrystals by flash Joule heating

**Fig. 1:**
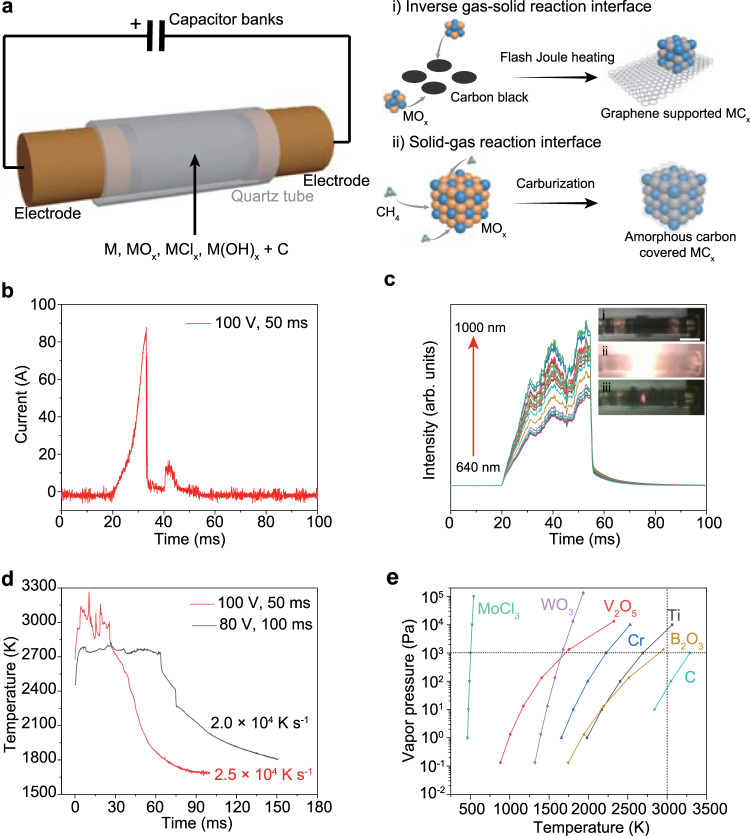
Ultrafast synthesis of carbides by flash Joule heating (FJH). **a** Schematic of FJH synthesis of carbides. Route (i) demonstrates the high-temperature FJH process disclosed here. Route (ii) demonstrates the traditional carburization process. **b** Current measurement during the FJH process. **c** Real-time spectral radiance at wavelength of 640–1000 nm. Inset, the pictures of the sample before FJH (i), during FJH (ii), and in rapid cooling (iii). Scale bar, 1 cm. **d** Real-time temperature measurement by fitting the blackbody radiation from the sample during the FJH process. **e** Temperature-vapor pressure relationships for various metal precursors and carbon. The vertical dashed line denotes temperature at 3000 K, and the horizontal dashed line denotes vapor pressure at 10^3^ Pa.

In a typical process, a mixture of metal precursors and commercial carbon black was slightly compressed inside a quartz tube between two graphite electrodes (Fig. 1a). The widely applicable metal precursors could be elemental metal (M), metal oxides (MO_x_), chlorides (MCl_x_), and hydroxides (M(OH)_x_), etc. The carbon black simultaneously works as the carbon source for carbothermic reduction and the conductive additive. The two electrodes were connected to a capacitor bank, which was first charged by a power supply and then brought the precursors to a high temperature by high voltage discharging (Supplementary Fig. 1). In a typical FJH process with a voltage of 100 V and a sample resistance of 1 Ω, the current passing through the sample was recorded to be ~100 A in ~50 ms discharge time (Fig. 1b).

A rapid light emission was observed during the FJH process (Fig. 1c, inset). The temperature was measured by fitting the blackbody radiation spectra of the sample (Fig. 1c, Supplementary Fig. 2). The highest temperatures obtained at 80 V and 100 V FJH were estimated to be ~2700 K and ~3000 K, respectively (Fig. 1d). The cooling rate is ultrafast and on the order of 10^4^ K s^−1^. The temperature distribution of the sample is simulated by using the finite element method (FEM) (Supplementary Note 1), which further provides insight into the effects of FJH parameters on the reachable temperature. It is found that higher temperature values could be obtained by applying a larger FJH voltage and suitable sample electrical conductivity (Supplementary Fig. 3). In contrast, a higher thermal conductivity of the sample results in a lower temperature due to a faster thermal dissipation. The temperature map shows that the temperature distribution is uniform throughout the entire sample (Supplementary Fig. 4), demonstrating the homogeneous heating feature of the FJH process.

FJH of the sample to such a high temperature (~3000 K) volatilizes most of the non-carbon components. According to the temperature-vapor pressure relationships (Fig. 1e), all of the representative metal precursors, including elemental metals and metal oxides and chlorides, have higher vapor pressure than carbon, which sublimes at ~3900 K^28^. As a result, the metal precursors are the volatile components, and the carbon source remains solid during the reaction. In this case, metal precursor vapors react with the carbon to form the metal carbides, which we define as the inverse gas-solid reaction interface (Fig. 1a, route i). In contrast, in the traditional carburization process^12^, a gaseous hydrocarbon such as methane (CH_4_) is introduced to the solid metal precursors. The carbon diffusion through the solid-gas interface is usually fast and results in a coked carbide surface due to the excessive supply of carbon sources (Fig. 1a, route ii), which can passivate the catalytic activity of the final products^7^.

### Phase controlled synthesis of molybdenum carbide nanocrystals

**Fig. 2:**
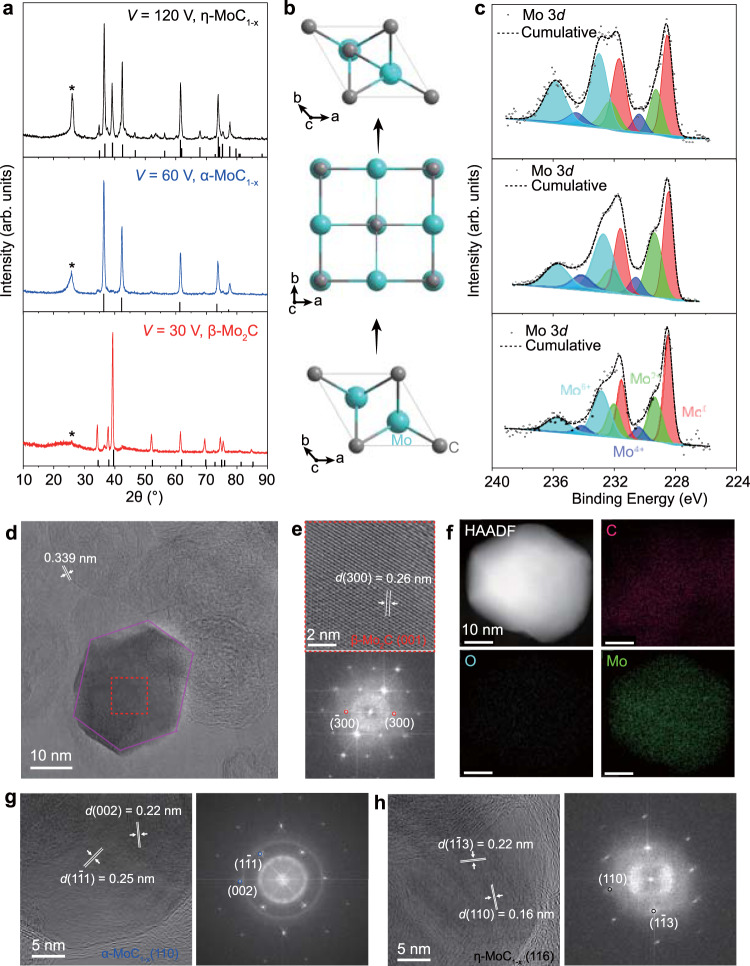
Phase controlled synthesis of molybdenum carbide. **a** X-ray diffraction (XRD) patterns of β-Mo_2_C, α-MoC_1-x_, and η-MoC_1-x_ synthesized at voltage (*V*) of 30 V, 60 V, and 120 V, respectively. The PDF reference cards for each are β-Mo_2_C, 35–0787; α-MoC_1-x_, 65–8092; and η-MoC_1-x_, 08–0384. The peak at ~26° (star) is attributed to graphene support. **b**, Crystal structures of three phases of molybdenum carbides. β-Mo_2_C is hexagonal with ABAB stacking (bottom), α-MoC_1-x_ is cubic (middle), and η-MoC_1-x_ is hexagonal with ABCABC stacking (top). **c** X-ray photoemission spectroscopy (XPS) spectra of three phases of molybdenum carbides. **d** Bright-field transmission electron microscopy (BF-TEM) image of a β-Mo_2_C nanocrystal supported on graphene. The 0.339 nm corresponds to interplanar distance (*d*) of graphene. The purple hexagon depicts the shape of the nanocrystal. **e** High-resolution transmission electron microscopy (HRTEM) image of β-Mo_2_C (top) and corresponding fast Fourier transform (FFT) pattern (bottom). **f** High-angle annular dark-field scanning transmission electron microscopy (HAADF-STEM) image and energy dispersive spectroscopy (EDS) element maps of β-Mo_2_C. **g** HRTEM image of α-MoC_1-x_ (left) and corresponding FFT pattern (right). **h**, HRTEM image of η-MoC_1-x_ (left) and corresponding FFT pattern (right).

We first tried to synthesize molybdenum carbides that are attractive for catalysts^11,19,29,30^. The phases of molybdenum carbide are complex due to their temperature-, composition-, and vacancy-dependent stability^31^. Different phases have distinct geometric and electronic structures^27,32^, and the catalytically relevant phases are hexagonal β-Mo_2_C^19,30,33^, cubic α-MoC_1-x_^11,32,34^, and hexagonal η-MoC_1-x_^34^ (Supplementary Fig. 5). MoCl_3_ was chosen as the precursor because of its high vapor pressure (Fig. 1e). We found that three pure phases of molybdenum carbides could be selectively synthesized by adjusting FJH voltages (Fig. 2a, b). β-Mo_2_C phase was produced under the voltage of 30 V according to X-ray diffraction (XRD) (Fig. 2a, bottom); when the voltage was increased to 60 V, pure α-MoC_1-x_ phase was obtained (Fig. 2a, middle); further increasing the voltage to 120 V led to η-MoC_1-x_ (Fig. 2a, top). Note that the diffraction peak at ~26° is attributed to the graphene support (Supplementary Fig. 6). The phase transformation from hexagonal β-Mo_2_C to cubic α-MoC_1-x_ and then to hexagonal η-MoC_1-x_ is a newly found topotactic transition pathway, which is distinct from the previous report^19^, where the α-MoC_1-x_ is transformed to β-Mo_2_C after a ~24 h annealing at 850 °C, and η-MoC_1-x_ is only stabilized by using a NiI_2_ additive at a higher temperature.

To investigate the electronic structures, X-ray photoelectron spectroscopy (XPS) spectra of the Mo 3*d* core level was collected (Fig. 2c). Mo 3*d* spectra are split into 3*d*_3/2_ and 3*d*_5/2_ peaks. The peak fitting shows four chemical states of Mo in molybdenum carbides, including Mo^0^, Mo^2+^, Mo^4+^, and Mo^6+^. The dominant Mo^0^ peak and the smaller peak of Mo^2+^ are attributed to the molybdenum carbide due to the coexistence of Mo-Mo and Mo-C bonds in molybdenum carbides^19^. Mo^4+^ and Mo^6+^ are assigned to MoO_2_ and MoO_3_, respectively, due to the surface oxidation of molybdenum carbides when exposed to air^19,30^. The quantitative analysis of the ratios of Mo chemical states shows the high oxidation states (Mo^4+^ and Mo^6+^) in η-MoC_1-x_ are larger than those in β-Mo_2_C and α-MoC_1-x_ (Supplementary Fig. 7), indicating that β-Mo_2_C is the most oxidation resistant phase followed by α-MoC_1-x_.

The morphology characterization by scanning electron microscopy (SEM) shows the fine powder feature of all three carbide phases (Supplementary Fig. 8). The energy dispersive spectroscopy (EDS) mapping images show a uniform distribution of Mo and C (Supplementary Figs. 8, 9). Transmission electron microscopy (TEM) and XRD were used to characterize the size and crystallinity of the molybdenum carbides. The particle sizes of the molybdenum carbide phases are determined by the FJH voltages (Supplementary Fig. 10). The β-Mo_2_C synthesized at the lowest voltage has the largest average size of ~26.4 nm, followed by α-MoC_1-x_ (~21.2 nm) and η-MoC_1-x_ (~20.1 nm). The smaller particle size obtained under higher voltage could be attributed to the faster nucleation kinetics at higher temperature^35^. The particle size values measured by TEM match well with the crystal size values determined by XRD using the Halder-Wagner method (Supplementary Table 2), indicating the single-crystal feature of the synthesized carbide particles.

The typical bright-field TEM (BF-TEM) image of a β-Mo_2_C nanocrystal shows the regular hexagonal nanoplate with a lateral size of ~20 nm supported on carbon (Fig. 2d). The high-resolution TEM (HRTEM) image shows the lattice fringes (Fig. 2e, top), where the 0.26 nm interplanar spacing (*d*) corresponds to the (300) plane of β-Mo_2_C. According to the atomic-resolution image and corresponding fast Fourier transform (FFT) pattern (Fig. 2e, bottom), the nanoplate orientation is assigned to β-Mo_2_C(001). The high-angle annular dark-field (HAADF) scanning transmission electron microscopy (STEM) image and EDS elemental maps under STEM mode reveal the uniform spatial distribution of Mo, C, and O (Fig. 2f). Note that the O is attributed to the surface contamination, consistent with the XPS results (Fig. 2c). The HRTEM image and corresponding FFT pattern of α-MoC_1-x_ (Fig. 2g, Supplementary Fig. 11) and η-MoC_1-x_ (Fig. 2h, Supplementary Fig. 12) are also obtained with the orientation of α-MoC_1-x_(110) and η-MoC_1-x_(116) for the specific samples. Nevertheless, no preferred orientation is observed for these carbide nanocrystals according to XRD results (Fig. 2a).

### Phase transformation process of molybdenum carbides revealed by ab initio calculations

**Fig. 3:**
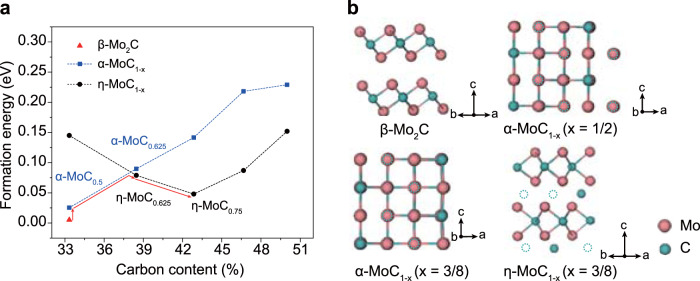
Phase transformation process of molybdenum carbides revealed by density functional theory (DFT) calculations. **a** Formation energy of β-Mo_2_C, and α-MoC_1-x_ and η-MoC_1-x_ with different carbon atomic content. The red line denotes the projected phase transformation pathway. **b** Calculated crystal structures of β-Mo_2_C, α-MoC_1-x_ (*x* = 1/2), α-MoC_1-x_ (*x* = 3/8), and η-MoC_1-x_ (*x* = 3/8). The dashed circles denote the carbon vacancies.

To explain the voltage-dependent phase formation, we first recorded the current passing through the samples and the temperature under different FJH voltages (Supplementary Fig. 13a–c). A higher voltage leads to higher temperatures and energy inputs (Supplementary Fig. 13). The maximum temperatures at FJH voltages of 30 V, 60 V, and 120 V were measured to be 839 K, 1468 K, and 3242 K, respectively (Supplementary Fig. 14). The formation energies of β-Mo_2_C, α-MoC_1-x_, and η-MoC_1-x_ varied with carbon content were calculated by first-principles density functional theory (DFT) (Fig. 3a, Supplementary Fig. 15). It is found that the β-Mo_2_C phase is the most stable phase with the lowest formation energy; hence, β-Mo_2_C forms at a relatively low voltage and temperature (Fig. 3a, Supplementary Fig. 14). In contrast, the α-MoC_1-x_ and η-MoC_1-x_ are metastable phases^31^ and are formed and stabilized at a higher temperature according to the Mo-C phase diagram. The α-MoC_1-x_ (*x* = 1/2) structure has a slightly higher formation energy and the same stoichiometric composition with β-Mo_2_C (Fig. 3b). Hence, the topotactic transition from β-Mo_2_C to α-MoC_1-x_ is expected when the carbon content is slightly increased (red arrow in Fig. 3a). As more carbon is incorporated into the Mo-C system, the α-MoC_1-x_ formation energy continuously increases, and the energy curve intersects with that of η-MoC_1-x_ (Fig. 3a). The η-MoC_1-x_ phase becomes the relatively stable phase near *x* = 3/8 (Fig. 3b), and continues to be the stable phase up to higher carbon contents. This result shows that the carbon vacancy dominates the energy landscape of the Mo-C system, and serves as the driving factor for the topotactic transition pathway from β-Mo_2_C to α-MoC_1-x_ and then to η-MoC_1-x_ phase.

The FJH process with broadly tunable energy input permits the access of the metastable phases with higher formation energy than the thermodynamically stable phase; then, the ultrafast cooling rate of the FJH process (>10^4^ K s^−1^) helps to kinetically retain the metastable phases, including α-MoC_1-x_ and η-MoC_1-x_ phases, to room temperature. As a control, at the same temperature when the metastable α-MoC_1-x_ phase is produced by FJH, the synthesis using a conventional tube furnace with its slow cooling rate of ~10 K min^−1^ only produces the thermodynamically stable β-Mo_2_C phase (Supplementary Fig. 16). This explicitly demonstrates the critical role of the ultrafast cooling rate of the FJH process in kinetically accessing the metastable phases.

### Phase dependent HER performance of molybdenum carbides

**Fig. 4:**
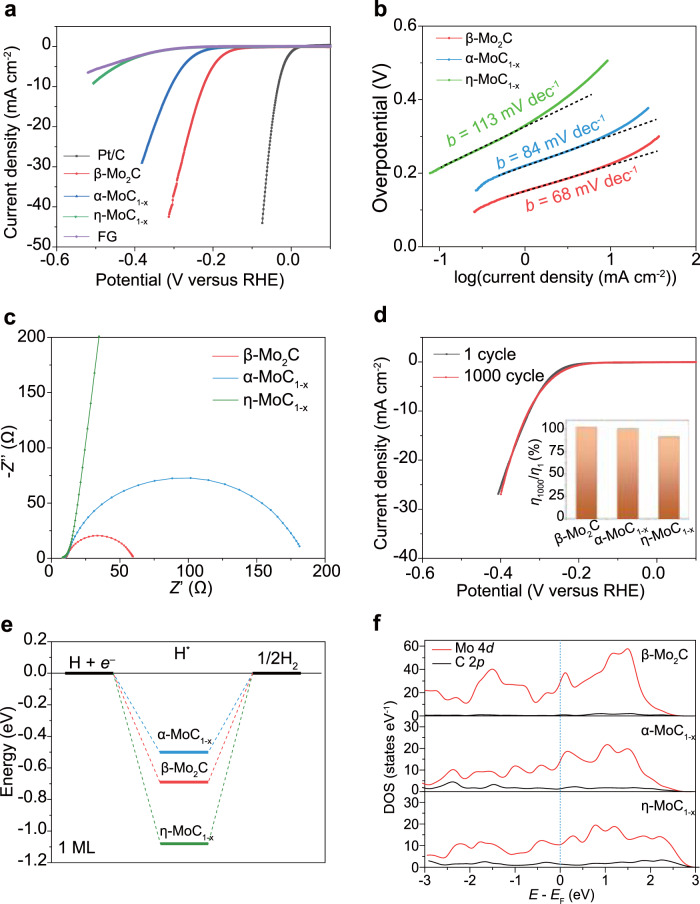
Phase dependent hydrogen evolution reaction (HER) performance of molybdenum carbides. **a** Polarization curves of three phases of molybdenum carbide. Pt/C and pure flash graphene (FG) were used as control. The performances were normalized to the same mass loading of molybdenum carbides. **b** Tafel curves of three phases of molybdenum carbide. **c** Alternating current (AC) impedance of three phases of molybdenum carbide. **d** Durability of molybdenum carbides showing the polarization curve of α-MoC_1-x_ for the 1st cycle and the 1000th cycle. Inset is the ratio of overpotentials at 1st cycle and 1000th cycle for three phases of molybdenum carbide. **e** Free-energy diagrams for HER on the β-Mo_2_C(001), α-MoC_1-x_(110), and η-MoC_1-x_(001) at one monolayer hydrogen adsorption coverage. **f** Calculated partial density of states of Mo and C in β-Mo_2_C(001), α-MoC_1-x_(110), and η-MoC_1-x_(001). The blue dashed line denotes the position of the Fermi level.

The side-by-side electrochemical comparison of the three phases of molybdenum carbide reveals the effect of the phase control on their individual intrinsic characteristics and catalytic behaviors. To demonstrate their catalytic properties, the HER performances of the three molybdenum carbide phases were measured in 0.5 M H_2_SO_4_ using a standard three-electrode configuration. Linear scan voltammogram (LSV) curves of the different electrocatalysts, as well as the Pt/C benchmark are shown in Fig. 4a. The flash graphene (FG) obtained from FJH of carbon black was used as a control and showed negligible HER activity^36^. The phase-dependent HER activity of molybdenum carbides was observed. The overpotential (*η*) versus a reversible hydrogen electrode (RHE) at geometric current densities of 10 mA cm^−2^ for β-Mo_2_C, α-MoC_1-x_, and η-MoC_1-x_ are –220 mV, –310 mV, and –510 mV, respectively (Fig. 4a). The Tafel slopes (*b*) for β-Mo_2_C, α-MoC_1-x_, and η-MoC_1-x_ are calculated to be 68 mV dec^−1^, 84 mV dec^−1^, and 113 mV dec^−1^, respectively (Fig. 4b), showing the phase-dependent HER reaction kinetics. The fast electrode kinetics of β-Mo_2_C phase is reflected in the small charge transfer resistance of ~60 Ω at the potential of –0.5 V versus RHE according to the electrochemical impedance measurement (Fig. 4c). The durability of the three molybdenum carbides phases was evaluated by sweeping the electrocatalysts for 1000 cycles using the cyclic voltammetry method. The LSV curves of the 1st and 1000th cycle for the three phases of molybdenum carbides are shown in Fig. 4d and Supplementary Fig. 17. No obvious current degradation was observed for all three phases, and the overpotential at 10 mA cm^−2^ declined little (Fig. 4d, inset), demonstrating the excellent long-term stability.

DFT calculations were conducted to elucidate the phase-dependent HER performance. The Gibbs free energy of hydrogen adsorption (Δ*G*_H_) has been a descriptor in the selection of HER electrocatalysts^37^, and optimal catalysts have Δ*G*_H_ near 0 eV according to the Sabatier principle^38^. The Δ*G*_H_ of β-Mo_2_C(001), α-MoC_1-x_(110), and η-MoC_1-x_(001) were calculated to be 0.48 eV, 0.71 eV, and 1.09 eV, respectively (Fig. 4e). These results show that β-Mo_2_C and α-MoC_1-x_ have smaller hydrogen adsorption energies than η-MoC_1-x_, consistent with previous reports^33,39^. Other than Δ*G*_H_, the electronic structures provide valuable insights into the metallic character of carbide phases^27^. Figure 4f illustrates the partial density of states (DOS) of Mo and C in molybdenum carbides. The DOS of β-Mo_2_C near the Fermi level is substantially larger than those of α-MoC_1-x_ and η-MoC_1-x_. The higher Mo content in β-Mo_2_C results in a higher carrier density and enhanced metallicity, which is beneficial for the charge transfer during electrochemical reactions (Fig. 4c). The larger surface area of β-Mo_2_C in comparison to the other two phases as measured by the Brunauer–Emmett–Teller (BET) method also contributes to the larger current density (Supplementary Fig. 18). The observed best HER performance of β-Mo_2_C is a collective effect of the relatively small hydrogen adsorption energy, enhanced metallic character, and high surface area. In addition, the flash graphene support provides a conductive pathway and prevents the carbide nanocrystals aggregating, which is beneficial for improving the HER performance^7,29^.

### Generalized strategy for carbide nanocrystals synthesis

**Fig. 5:**
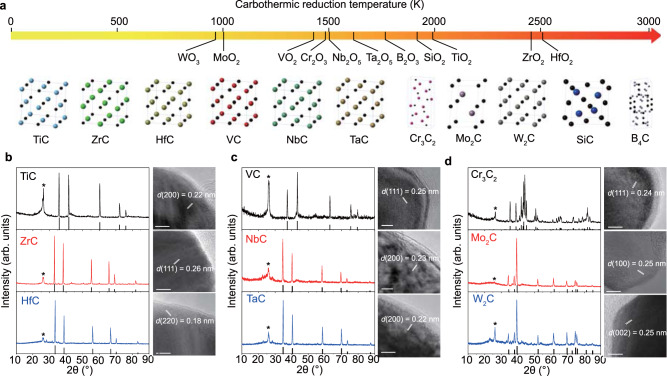
Generalized strategy for carbide synthesis. **a** Carbothermic reduction temperature of oxides derived from the Ellingham diagram, and the crystal structures of eleven carbides. **b** X-ray diffraction (XRD) patterns and high-resolution transmission electron microscopy (HRTEM) images of group IVB metal carbides. The PDF reference cards for each are TiC, 65–7994; ZrC, 65–8834; and HfC, 65–7326. **c** XRD patterns and HRTEM images of group VB metal carbides. The PDF reference cards for each are VC, 65–8825; NbC, 65–8780; and TaC, 65-0282. **d** XRD patterns and HRTEM images of group VIB metal carbides. The PDF reference cards for each are Cr_3_C_2_, 65–0897; Mo_2_C, 35–0787; and W_2_C, 20–1315. Scale bars are 5 nm in **b**, **c**, and **d**. The peak at ~26° (star) for all the samples is attributed to graphene support.

Because of the ultrahigh available temperature by the FJH process, various TMCs are readily synthesized regardless of the availability of metal precursors with high vapor pressure. A series of carbide nanocrystals from transition groups IVB, VB, and VIB were successfully synthesized (Fig. 5, Supplementary Table 2). The uniform temperature distribution permits the phase-pure synthesis throughout the entire sample (Supplementary Fig. 4). According to the Ellingham diagram, the reduction temperatures of the metal oxides were calculated, which serve as reference values to evaluate carbide formation since the reaction of metal with carbon is exothermic (Fig. 5a, Supplementary Fig. 19). The ultrahigh temperature (~3000 K) of the FJH process makes it possible for the reduction of all the listed oxides to elemental metals, including the most challenging HfO_2_ at temperature up to ~2510 K. Nearly all the low-cost metal or metal compounds, including oxides, hydroxides, and chlorides, could be used as precursors, making FJH a promising low-cost production method when compared to previous methods that rely on the availability of volatile compounds^18,22,23^.

Group IVB carbides only have the stable rock salt crystal structure, including TiC, ZrC, and HfC, which are readily synthesized (Fig. 5b). The particle sizes of the TiC, ZrC, and HfC were measured to be ~30.4 nm, ~38.6 nm, and ~30.6 nm, respectively (Supplementary Fig. 20). These values matched well with the crystalline sizes determined by XRD (Supplementary Table 2), demonstrating that the as-synthesized carbide nanoparticles are mostly single-crystal. For group VB carbides, the competing M_2_C (*M* = V, Nb, and Ta) phases could exist at a lower C content^31^. Nevertheless, by using a large molar ratio of C/M, we successfully synthesized the pure phases of VC, NbC, and TaC nanocrystals with the cubic structure and particle sizes ranging from 20 to 30 nm (Fig. 5c, Supplementary Fig. 21). In contrast, the phases of group VIB carbide (Cr, Mo, and W) are much more complex^40^. Here, the orthorhombic Cr_3_C_2_ phase and hexagonal W_2_C phase were synthesized with particle sizes of ~14.2 nm and ~18.7 nm, respectively (Fig. 5d, Supplementary Fig. 22). W_2_C is not thermodynamically favored over the WC phase below 1250 °C according to the W-C phase diagram^41^. The successful synthesis of the metastable W_2_C is attributed to the high energy input and ultrafast cooling rate of the ultrafast electrical thermal reaction, once again demonstrating the excellent phase engineering ability of the FJH process. Apart from the TMCs, the covalent carbides of B_4_C and SiC were synthesized (Supplementary Figs. 23, 24), further demonstrating the generality of the FJH process.

## Discussion

The as-synthesized carbide nanocrystals are supported on flash graphene. The necessity of the separation of graphene and carbides depends on the further application. For the application of nanocrystalline carbides in electrocatalysts, the graphene support is beneficial for improving the performance by providing conduction and preventing particle aggregation (Fig. 4). For another major application of nanocrystalline carbides as precursors for ultra-strong ceramics, the removal of excess carbon is necessary. Here, we realized the efficient purification of the carbides by post-synthesis processes (Supplementary Note 2), including the simple calcination in air^42^ for SiC (Supplementary Fig. 25); the Ca metal etching^43^ for TiC (Supplementary Figs. 26, 27), ZrC (Supplementary Fig. 28), HfC (Supplementary Fig. 29), VC (Supplementary Fig. 30), NbC (Supplementary Fig. 31), TaC (Supplementary Fig. 32), Cr_3_C_2_ (Supplementary Fig. 33), β-Mo_2_C (Supplementary Figs. 34, 35), and W_2_C (Supplementary Fig. 36); and the density-in-liquid purification procedure for metastable molybdenum carbides, α-MoC_1-x_ (Supplementary Fig. 37) and η-MoC_1-x_ (Supplementary Fig. 38). In addition, we also demonstrated the greatly improved purity of B_4_C by using controlled feeding during the synthesis (Supplementary Fig. 39).

Due to the ultrafast heating/cooling rate, the direct sampling heating feature, and the short reaction duration within 1 s, the FJH process for carbide synthesis is highly energy efficient compared to traditional furnace heating where large amounts of energy are used to maintain the temperature of the chamber. The carbide nanocrystals are synthesized at only 2.2–8.6 kJ g^−1^ in electrical energy (Supplementary Note 3). The FJH synthesis possesses excellent scalability, that a constant temperature value and uniformity on different mass scales could be obtained by adjusting the discharging voltage and/or the capacitance (Supplementary Note 4). We demonstrated the synthesis of carbide nanocrystals up to gram scale by increasing the FJH voltage (Supplementary Fig. 40). The FJH process is expected to be extended to the synthesis of carbide alloys^44^, heteroatom-decorated carbides^34^, and phase engineering of metastable carbides^45^, which provides a powerful technique for carbide production.

The controlled synthesis of metastable phases is challenging in the synthesis of inorganic materials^46^. The FJH process provides broadly tunable energy input that can exceed 3000 K coupled with a kinetically controlled ultrafast cooling rate (>10^4^ K s^−1^). Hence, the FJH process could provide access to many non-equilibrium phases and subsequently retain it at room temperature, thus serving as a potential tool for engineering the metastable phases of various materials, such as metal nanomaterials^46^, layered oxides^47^, metal nitrides^48^, and two-dimensional materials^49^.

## Methods

### Materials

The non-carbon precursors for carbide synthesis are Ti powder (Johnson Matthey, 99%), Zr(OH)_4_ powder (Sigma-Aldrich, 97%), HfO_2_ powder (Alfa Aesar, 99.95%), VO_2_ powder (Alfa Aesar, 99%), NbCl_5_ powder (Sigma-Aldrich, 99%), TaCl_5_ powder (Sigma-Aldrich, 99.8%), Cr powder (Alfa Aesar, APS < 10 μm, 99.2%), MoCl_3_ powder (Fisher Scientific, 99.5%), WO_3_ powder (Johnson Matthey, 99.998%), B powder (Alfa Aesar, 99.9%), and SiO_2_ powder (Sigma-Aldrich). Carbon black (Cabot, VULCANXC72R) is used at the carbon source as well as the conductive additive.

### FJH system and synthesis process

The electrical circuit diagram and setup of the FJH system are shown in Supplementary Fig. 1a, b. A capacitor bank with a total capacitance of 60 mF was used as the power supply. The metal precursors and carbon black with specific weight ratios (Supplementary Table 2) were mixed by grinding using a mortar and pestle. The reactants (~50 mg) were loaded into a quartz tube with an inner diameter (ID) of 4 mm and outside diameter (OD) of 8 mm. When scaling up the process, a quartz tube with ID of 8 mm and OD of 12 mm was used for the ~200 mg sample, and a quartz tube with ID of 16 mm and OD of 20 mm was used for the ~1 g sample. Graphite rods were used as the electrodes in both ends of the quartz tube. The electrodes were loosely fitting in the quartz tube to permit outgassing. The resistance was controlled by the compression force of the electrodes across the sample. The tube was then loaded on the reaction stage (Supplementary Fig. 1c). The reaction stage was loaded into a sealed reaction chamber which was evacuated to a mild vacuum (~10 mm Hg) to accommodate degassing and avoid sample oxidation (Supplementary Fig. 1d). The reaction stage was then connected to the FJH system. The capacitor bank was charged by a direct current (DC) supply that can reach voltages up to 400 V. A relay with programmable ms-level delay time was used to control the discharge time. The charging, flash Joule heating, and discharging were automatically controlled by using the National Instruments Multifunction I/O (NI USB-6009) combined with a customized LabView program. After the FJH reaction, the apparatus rapidly cooled on its own to room temperature. Before removing the sample, make sure that the capacitor bank is fully discharged. The detailed conditions for the synthesis of various carbides are listed in Supplementary Table 2. CAUTION: There is a risk of electrical shock if improperly operated. The recommended safety practices were listed in the Supplementary Information.

### Characterization

The SEM images and element maps by EDS were obtained using a FEI Helios NanoLab 660 DualBeam SEM system at 5 kV. The Raman spectra were acquired using a Renishaw Raman microscope (laser wavelength of 532 nm, laser power of 5 mW, lens of 50 X). XRD patterns were collected by using a Rigaku D/Max Ultima II system configured with a Cu Kα radiation (*λ* = 1.5406 Å) source. The Halder-Wagner method was used for the crystal size determination. XPS analyses were conducted using a PHI Quantera XPS system under the base pressure of 5 × 10^−9^ Torr. Elemental spectra were collected using a step size of 0.1 eV with the pass energy of 26 eV. All the XPS spectra were calibrated by using the standard C 1 *s* peak at 284.8 eV. BF-TEM and HRTEM images were taken on a JEOL 2100 field emission gun transmission electron microscope under the voltage of 200 kV. STEM, HAADF, and EDS maps were obtained by using the FEI Titan Themis3 system equipped with image and probe aberration corrections and an electron monochromator at 80 kV. BET measurements were carried out on a Quantachrome Autosorb-iQ3-MP/Kr BET Surface Analyzer, and nitrogen was used as the adsorption/desorption gas.

### Temperature measurement

The temperature was measured by fitting the blackbody radiation of the sample during FJH using a homemade spectrometer (Supplementary Fig. 2). The spectral radiance from the sample was collected by a 16-channel photomultiplier tube array in the wavelength ranges of 640–1000 nm. The emission spectra were then fitted to the blackbody radiation by using the Eq. (1),

1 $${B}_{\lambda }\left(\lambda ,T\right)=\gamma \frac{2h{c}^{2}}{{\lambda }^{5}}\frac{1}{{e}^{{hc}/\lambda {k}_{B}T}-1}$$

where *B*_*λ*_ is the radiance, *λ* is the wavelength, *T* is the fitted temperature, *γ* is a constant introduced for fitting, *h* is the Planck constant, *c* is the speed of light, and *k*_B_ is the Boltzmann constant. Prior to measure the sample, a 2800 K lamp was used to calibrate the temperature. The temperature distribution was assessed based on the optical images of the sample during FJH taken using an ultrafast camera (Chronos 1.4) and fitting of the Stefan-Boltzmann law as in Eq. (2),

2 $${j}^{* }=\sigma {T}^{4}$$

where j^*^ is the blackbody radiant emittance, *σ* is a constant of proportionality, and *T* is the thermodynamic temperature.

### HER test

The binder solution was prepared by mixing of 5 wt% Nafion solution (80 μL) with water:ethanol (1 mL, 1:1, volume ratio). The catalyst ink was then prepared by dispersion of molybdenum carbides (1 mg, or flash graphene) into binder solution (1 mL) followed by ultrasonication for 1 h. The ink (40 μL) was then dropcast onto a glassy carbon electrode with a diameter of 3 mm (catalyst loading ~0.57 mg cm^−2^). Electrochemical measurements were conducted using a CHI 608D electrochemical workstation in a 0.5 M H_2_SO_4_ solution. The standard three-electrode setup was applied, where a saturated calomel electrode (SCE) was used as the reference, the catalyst-loaded glassy carbon electrode was used as the working electrode, and a graphite rod was used as the counter electrode. Before measurement, the electrolyte was purged with Ar gas for Ar saturation. Linear sweep voltammetry (LSV) was carried out at a scan rate of 5 mV s^−1^. To compare the intrinsic properties of the three molybdenum carbide phases, the electrocatalytic performances are normalized to the same mass loading of molybdenum carbides (~0.074 mg cm^−2^), and the trend remains the same, that β-Mo_2_C has the best performance, followed by α-MoC_1-x_, and then η-MoC_1-x_ (Fig. 4). Electrochemical impedance tests were carried out in the frequency range of 100000 to 0.05 Hz.

### DFT calculation

DFT methods^50^ were used as they are implemented in the Vienna Ab-initio Simulation Package (VASP)^51^. A plane wave expansion up to 500 eV was employed in combination with an all-electron-like projector augmented wave (PAW) potential^52^. Exchange-correlation was treated within the generalized gradient approximation (GGA) using the functional parameterized by Perdew, Burke, and Ernzerhof^53^. For calibration, we have calculated the three molybdenum carbides, graphite, and body-centered cubic (BCC) molybdenum metal bulk structures. Periodic boundary conditions were applied to the unit cell in all three dimensions. The Brillouin zone integrations were performed using Monkhorst-Pack type meshes^54^, with sufficient meshes of *k*-points chosen so that the energy and lattice constant were fully converged. All structures were considered to be fully relaxed when the maximum force on each atom is smaller than 0.01 eV Å^−1^. The calculated lattice constant of the BCC Mo metal is 3.170 Å. The lattice constants of graphite are *a* = 2.467 Å and *c* = 7.884 Å. The rock-salt structure of α-MoC has the lattice constant of *a* = 4.383 Å. The hexagonal β-Mo_2_C has lattice constants of *a* = 3.054 Å and *c* = 4.816 Å, and hexagonal η-MoC has lattice constants of *a* = 3.025 Å and *c* = 10.579 Å.

The pristine α-MoC(110), β-Mo_2_C(001), η-MoC(001) surfaces were modeled using slabs with the thickness of four bilayers (13 Å), four tri-layers (13 Å), and four quadra-layers (20 Å), respectively. Each slab has two equivalent surfaces without reconstruction except for η-MoC(001). This is because the (001) cleavage plane of the η-MoC goes between the intercalated carbon layer and a Mo_2_C tri-layer. Therefore, such a cleavage results in asymmetric surfaces of one with a monolayer of C adatoms, and the other with no C adatoms. This creates a long-range dipole between the two sides of the slab, which is energetically unfavorable. To create a slab with symmetric surfaces, we divide the C adatoms into the two surfaces so that each surface has half a monolayer of C adatoms. The vacuum layer between the slabs is chosen to be 10 Å or thicker. Hydrogen adsorption to the surfaces is studied by first placing a single H atom at all possible nonequivalent adsorption sites to determine the most favorable H location. Then H atoms are placed to all the equivalent sites. The adsorption energy per H atom is calculated with respect to the pristine surface and reservoir of free hydrogen molecules. We do not include the entropy here because it depends on the condition of operation.

Finally, to study the formation energy of α-MoC_1-x_ and η-MoC_1-x_ phases with respect to the C content, we started with 2 × 2 × 2 supercells of the C-saturated α-MoC and η-MoC crystals. Then step by step, 1/8 of the C atoms were removed per step and the structures were relaxed. The size of the supercell was also relaxed with preserved symmetry. In each step, the removal of different C atoms creates different structures of vacancies. Therefore, all possible structures of MoC_1-x_ must be calculated for a fixed *x* to find the lowest-energy structure. For example, in η-MoC phase, removal of the intercalated C atoms surprisingly cost more energy than removal of the C atoms in the tri-layer. The formation energy is calculated using the following Eq. (3)^40^,

3 $${E}_{{{{{{\rm{form}}}}}}}=\frac{E\left({{{{{{\rm{Mo}}}}}}}_{{{{{{\rm{m}}}}}}}{{{{{{\rm{C}}}}}}}_{{{{{{\rm{n}}}}}}}\right)-{mE}\left({{{{{{\rm{Mo}}}}}}}_{{{{{{\rm{bcc}}}}}}}\right)-{nE}({{{{{{\rm{C}}}}}}}_{{{{{{\rm{graphite}}}}}}})}{n+m}$$

where *n* and *m* are the number C and Mo atom in the supercell of a MoC_1-x_ structure with a total energy *E*(Mo_m_C_n_), *E*(Mo_bcc_) is the energy per Mo atom in its BCC bulk crystal, and *E*(C_graphite_) is the energy per C atom in graphite.

## Supplementary information

Supplementary Information

## Data Availability

The data supporting the findings of this study are available within the article and its Supplementary Information. Other relevant data are available from the corresponding author upon reasonable request. The source data generated in this study have been deposited in the Zenodo database under 10.5281/zenodo.5687314. Source data are provided with this paper.

## Source data

Source Data
