# Supplementary material for: Phase controlled synthesis of transition metal carbide nanocrystals by ultrafast flash Joule heating
Source: Nat Commun. 2022 Jan 11;13:262. doi: 10.1038/s41467-021-27878-1 (PMC8752793; doi:10.1038/s41467-021-27878-1)
Supplement: Supplementary file 1 — Supplementary Information [file 41467_2021_27878_MOESM1_ESM.pdf]

Supplementary Information

Phase controlled synthesis of transition metal carbide nanocrystals by ultrafast flash Joule heating

B. Deng et al.

Supplementary Note 1. Simulation of the temperature distribution.

The temperature distribution was simulated based on the finite element method (FEM) using the COMSOL Multiphysics 5.5 software. The Joule heating mode in the AC/DC module was used. The geometric configuration, materials properties, and boundary conditions are listed in **Supplementary Table 1**. Similar to the real reaction tube, a cylinder is used as the geometric configuration with electrode radius (0.2 cm), electrode length (0.5 cm), materials length (2 cm), and materials radius (0.2 cm). The electrical conductivity (σ) and thermal conductivity (κ)[1] of the carbon black are from the literature reported values.

The temperature distribution of the sample is shown in **Supplementary Fig. 2a**. The simulated sample temperature is up to ~3000 K at the center, while the sample temperature at the edge is slightly lower than that at the center because of thermal dissipation at the sample-electrode interface[2]. The simulated temperature values match well with the measured values by fitting the blackbody radiation (**Fig. 1d**). The temperature simulation further provides insight into the effects of Joule heating parameters on the available temperature. We identified that the Joule heating voltage, electrical conductivity of the materials, and thermal conductivity of the materials are critical parameters. We found that a higher voltage leads to higher temperature (**Supplementary Fig. 2b**, **Supplementary Fig. 14**). Also, a better sample electrical conductivity leads to higher temperature (**Supplementary Fig. 2c**). In contrast, a higher thermal conductivity of the sample results in lower temperature due to thermal dissipation (**Supplementary Fig. 2d**).

Supplementary Note 2. Removal of graphene and purification of the carbides.

There have been several post-synthesis purifications processes to remove excess carbon from carbides, including oxidization[3], H_2 etching[4,5], and Ca metal reaction[6]. The purification process depends on the properties of the specific carbide. For example, for SiC with higher resistance to oxidation than graphene, it is possible to remove carbon by just calcination in air[3]. In contrast, the simple calcination process is not suitable for TMCs since they are oxidized prior to graphene. Hence, for TMCs, we here demonstrated a Ca metal reaction process[6] for purification.

Purification of SiC by calcination in air.

It is reported that SiC could withstand a high temperature up to 921 °C in air[3]. As shown in **Supplementary Fig. 25a**, the XRD pattern of the as-synthesized mixture of SiC/graphene shows a graphite peak at ~26°. The TGA curve of the mixture of SiC/graphene conducted in air shows a monotonic decrease from ~550 °C to ~750 °C (**Supplementary Fig. 25b**). Hence, we calcined the samples at 800 °C in air for 30 min. The XRD pattern of the product show a pure phase of SiC without any peaks from graphitized carbon or amorphous carbon (**Supplementary Fig. 25c**). The Raman spectrum of the as-synthesized mixture of SiC/graphene shows characteristic D, G, and 2D bands of graphene (**Supplementary Fig. 25d**). Notably, after purification, no graphene peak was identified (**Supplementary Fig. 25d**). Considering the excellent resolution of Raman for detecting even monolayer graphene, this result demonstrates the efficient removal of the excess carbon.

Purification of TMCs by Ca metal reaction.

The TMC nanocrystals were purified by a Ca metal reaction process[6]. We first used the mixture of TiC/graphene as an example to discuss the purification process in detail, and then provide the purification results for the other TMCs.

The as-synthesized mixture of TiC/graphene (~20 mg) was placed into a stainless steel crucible, and Ca metal (~80 mg) was added. Then, the crucible was loaded into a tube furnace (Thermo Scientific, Lindberg tube furnace). Ar gas with the flow of ~400 sccm was used as the inert gas under atmospheric pressure (AP). The temperature was ramped to 860 °C (ramping rate of ~40 °C min^{-1}), which is somewhat above the melting point of Ca (839 °C), and maintained for 10 min. The excess carbon reacts with Ca with the following reaction,

$$Ca + 2C = CaC_2 \quad (1)$$

Then, the furnace was cooled to room temperature. The resulted gray solid was placed into water to remove the CaC_2 and Ca residues by the following reactions,

$$CaC_2 + H_2O = Ca(OH)_2 + C_2H_2 \quad (2)$$

$$Ca + H_2O = Ca(OH)_2 + H_2 \quad (3)$$

The undissolved TiC particles were dried for characterization (**Supplementary Figs. 26-27**).

As shown in **Supplementary Fig. 26a**, the as-synthesized mixture of TiC/graphene shows a strong peak at ~26°, which is from the graphene support; after purification, no graphitized or amorphous carbon peak was identified. The Raman spectrum of the as-synthesized mixture of TiC/graphene show characteristic D, G, and 2D bands of graphene (**Supplementary Fig. 26b**). Notably, after purification, no graphene peak is identified. Considering the excellent resolution of Raman spectroscopy for detecting even monolayer graphene, this result demonstrated the efficient removal of the excess carbon. The XPS analyses confirm the chemical states of Ti and C in TiC (**Supplementary Figs. 26c-d**). Note that the Ti-O could be due to the surface oxidation, and the

C-C peak could be from the absorbed hydrocarbon in air. The BF-TEM image shows no excess carbon on the purified TiC nanoparticles (**Supplementary Fig. 27a**). Moreover, the particle size is measured to be ~35.1 nm according to the TEM statistics (**Supplementary Fig. 27b**), which is slightly larger than that of the as-synthesized particles (~30.4 nm, **Supplementary Figs. 20a-b**), demonstrating that the purification process did not result in significant coarsening of the nanoparticles. The HRTEM images show the clean surface of the purified TiC nanoparticles without any carbon layer coating (**Supplementary Figs. 27c-d**), further demonstrating the efficient removal of all the excess carbon.

By using the same procedure, we have purified most of the synthesized TMCs nanocrystals, including TiC (**Supplementary Figs. 26-27**), ZrC (**Supplementary Fig. 28**), HfC (**Supplementary Fig. 29**), VC (**Supplementary Fig. 30**), NbC (**Supplementary Fig. 31**), TaC (**Supplementary Fig. 32**), Cr_3C_2 (**Supplementary Fig. 33**), β-Mo_2C (**Supplementary Fig. 34-35**), and W_2C (**Supplementary Fig. 36**).

Nevertheless, we found that the Ca reaction method is not suitable for three carbides: B_4C, α-MoC_{1-x}, and η-MoC_{1-x}. For B_4C, we found that after the same process, no residual solid remained. This is probably due to the reaction of B_4C with Ca at high temperature. For α-MoC_{1-x} and η-MoC_{1-x}, the purification process could indeed remove the excess carbon (**Supplementary Fig. 35**). However, we found that the α-MoC_{1-x} and η-MoC_{1-x} phases were transformed to β-Mo_2C after purification (**Supplementary Fig. 35**). As we discussed in detail in the manuscript (**Fig. 3**), β-Mo_2C is the thermodynamically stable phase, while the α-MoC_{1-x} and η-MoC_{1-x} phases are metastable phases. The α-MoC_{1-x} and η-MoC_{1-x} phases are successfully synthesized and stabilized to room temperature due to the ultrafast cooling rate (~10^4 °C s^{-1}) of the FJH process. For the Ca reaction purification process, we used a CVD furnace, and the cooling rate was low (~10 °C min$^-$

[1]). As a result, the metastable α-MoC_{1-x} and η-MoC_{1-x} phases were degraded to the thermodynamically stable β-Mo_2C phase.

Purification of metastable molybdenum carbides by a physical process.

For the cases that the above chemical separation processes are not applicable, including α-MoC_{1-x} and η-MoC_{1-x}, we here applied the physical separation approach to remove excess carbon. Experimentally, the mixture of graphene and carbides were dispersed into dimethylformamide (DMF) solvent, and cup-horn ultrasonicated for 1 h. Then, the dispersion was centrifuged to precipitate the carbide solid, while the graphene mostly remained dispersed in the supernatant. The supernatant was removed, and the precipitate was purified again by the cycle of dispersion-ultrasonication-centrifugation process. After 5 to 6 cycles, the supernatant was clear, indicating that all the dispersible free carbons were removed. The obtained solids were dried in an oven (~100 °C).

The results for α-MoC_{1-x} were shown in **Supplementary Fig. 37**. According to the XRD (**Supplementary Fig. 37a**), the graphene peak (*) intensity is greatly reduced after purification even though it is not completely removed. By using the XRD peak intensity ratio of carbide and graphene as an index, the removal efficiency of the excess carbon is ~80% by using the physical purification process. The Raman spectra similarly show the removal of carbon. Before purification, the carbides were covered by graphene and hence the carbide peaks were undetectable (**Supplementary Fig. 37b**). In contrast, after purification, the carbide bands were clearly seen. The XPS confirms the chemical states of Mo and C in α-MoC_{1-x} (**Supplementary Figs. 37c-d**). The Mo^0 and Mo^{2+} are from the molybdenum carbide phase, where the Mo^{4+} and Mo^{6+} are from the surface oxide, as we have discussed in detail in the manuscript (**Fig. 2c**). The results for η-

MoC_{1-x} are shown in **Supplementary Fig. 38**, which is similar with that of the α-MoC_{1-x}. According to the change of XRD peak intensity ratio of graphene and carbide, the removal efficiency of graphene is ~60% for the η-MoC_{1-x}. The remaining carbon might be chemically bonded with carbide and hence is difficult to remove by the physical purification process.

Improving the purity of B_4C by changing the precursor feeding ratio.

B_4C is another carbide that is difficult to be purified by the chemical processes. B_4C is more susceptible than carbon, and hence is unable to be purified by the chemical oxidation process. In addition, we also found that the Ca metal etching process is not suitable for B_4C because it is reactive with Ca metal at high temperature. Here, we provided the strategy for the production of high-purify B_4C through a synthetic modification.

According to the stoichiometry of B_4C, the mass ratio of B and C is m(B):m(C) = 3.6:1. In our previous synthesis, we used the mass ratio of m(B):m(C) = 1:2, and the resulting content of B_4C in the product was c(B_4C) ~49 wt% (**Supplementary Fig. 39a**). Here, we used the stoichiometric feeding of m(B):m(C) = 3.6:1, and the content of B_4C was improved to ~95 wt% (**Supplementary Fig. 39a**). The Raman spectrum of the sample synthesized at m(B):m(C) = 1:2 also shows the graphene bands (D and G) with the bands from B_4C (**Supplementary Fig. 39b**). In contrast, the sample synthesized at stoichiometric feeding of m(B):m(C) = 3.6:1 show only carbide bands in most regions (**Supplementary Fig. 39b**).

Supplementary Note 3. Electrical energy consumption for the synthesis of carbide by FJH.

The energy consumption is calculated by Supplementary Equation 4,

$$E = \frac{(V_1^2 - V_2^2) \times C}{2 \times M} \qquad (4)$$

where E is the energy per gram (kJ g^{-1}), V_1 and V_2 are the voltage before and after flash Joule heating, respectively, C is the capacitance (C = 60 mF), and M is the mass per batch.

By using the upper bounds of V_1 = 120 V, V_2 = 0 V, M = 0.05 g, the energy is calculated to be,

E = 8.6 kJ g^{-1} = 2.39 kWh kg^{-1}

By using the lower bounds of V_1 = 30 V, the energy was calculated to be,

E = 2.2 kJ g^{-1} = 0.61 kWh kg^{-1}

Hence, the energy used to synthesize the carbide is in the range of 2.2 – 8.6 kJ g^{-1}.

Given that the industrial price of electric energy in Texas, USA is \$0.02 kWh^{-1}, the cost for synthesis of carbides would be P = 0.012 – 0.048 \$ kg^{-1}.

Supplementary Note 4. Strategies for scaling up of the FJH process.

Since the reaction temperature is critical for carbide synthesis, the scaling-up of the synthesis by FJH requires that the temperature value and uniformity remain the same at different mass scale. Here, we firstly derived the key parameters of the FJH dominating the sample temperature, and then experimentally demonstrated the scaling up of the FJH process.

The charge (q) in the capacitor banks is determined by Supplementary Equation 5,

$$q = CV \qquad (5)$$

where C is the total capacitance of the capacitor bank, and V is the charging voltage.

The average current (I) is calculated by Supplementary Equation 6,

$$I = \frac{q}{t} \qquad (6)$$

where t is the discharging time, supposing that the charges in the capacitor are totally discharged in the discharge time.

As a result, the current density (j) is calculated by Supplementary Equation 7,

$$j = \frac{I}{S} = \frac{CV}{St} \qquad (7)$$

where S is the cross-sectional area of the sample.

Since we use a cylinder-shaped tube and sample, so the mass (m) of the sample is calculated by Supplementary Equation 8,

$$m = \rho S l \qquad (8)$$

Where ρ is the density of the sample, l is the thickness of the sample.

Above all, we get the Supplementary Equation 9,

$$j = \frac{CV\rho l}{mt} \qquad (9)$$

According to the Joule heating law, the heat amount (Q) per volume is determined by Supplementary Equation 10,

$$Q \propto j^2/\sigma \qquad (10)$$

where σ is the electrical conductivity of the sample.

To realize the same reaction condition for different sample mass, we should maintain a constant heat amount (Q) per volume and a constant short FJH time (t). Since σ, ρ are constant for the specific sample, according to Supplementary Equation 9 and Supplementary Equation 10, when the mass (m) is increased, we could either increase the FJH voltage (V), or increase the capacitance (C) of the capacitor banks, to realize a constant temperature value and distribution.

We demonstrated the scalable synthesis of metal carbide by FJH to gram scale. As shown in **Supplementary Fig. 40a**, we used three quartz tubes with the diameters of 0.4 cm, 0.8 cm, and 1.6 cm for the synthesis of TiC nanocrystals with the mass of ~50 mg, ~200 mg, and ~1 g. We here used the increasing voltage strategy. As shown in **Supplementary Fig. 40b**, the XRD patterns show the successful synthesis of pure TiC phase for all three samples at different mass scale.

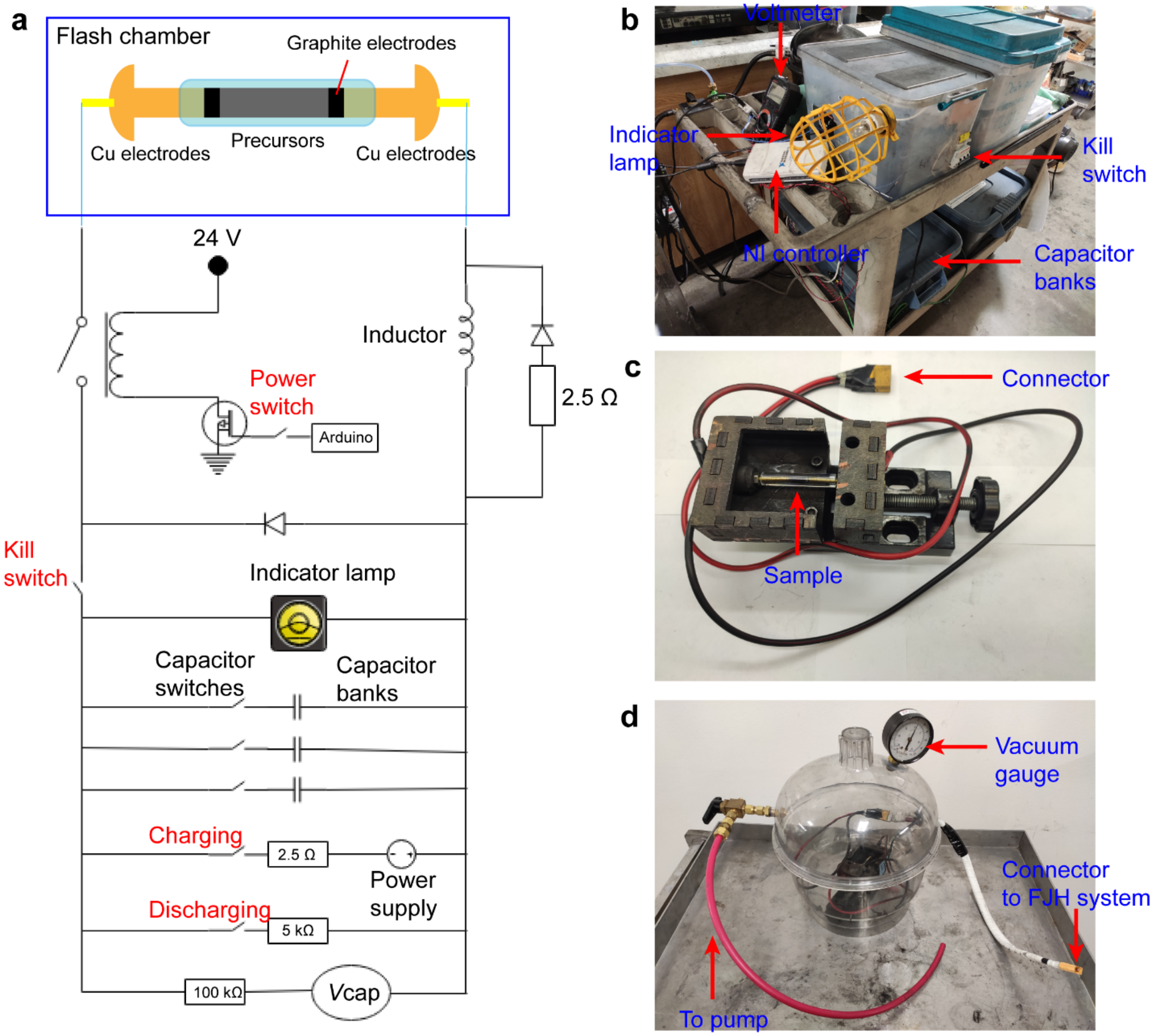

Supplementary Fig. 1. The flash Joule heating (FJH) setup. (**a**) Electrical schematic of the FJH system. A capacitor bank composed of 10 aluminum electrolytic capacitors (450V, 6 mF, Mouser #80-PEH200YX460BQU2) with a total capacitance of 60 mF is used for charging. Additional details of the electrical components could be found in our previous publication[7]. (**b**) Picture of the FJH setup. (**c**) Picture of the reaction stage. (**d**) Picture of the reaction chamber.

CAUTION: There is a risk of electrical shock if improperly operated. The below safety guidelines are recommended[7]:

(1) Enclose or carefully insulate the wire connections.

(2) All connections and wires must be suitable for high voltages and currents.

(3) Keep in mind that the system can discharge thousands of Joules in milliseconds, which could cause components such as relays to explode.

(4) Keep a voltmeter with high voltages test available at all times. When working on the capacitor bank, always check the voltage on each.

(5) Safety glasses for welding are recommended to block the infrared and ultraviolet light during the flashing reaction.

(6) One hand rule should be obeyed to prevent electrocution: use only one hand when working on the system, with the other hand not touching any grounded surface.

(7) Thick rubber gloves extending to the elbows should be worn.

(8) The reliability and robustness of the system should be confirmed by an experienced electrical technician.

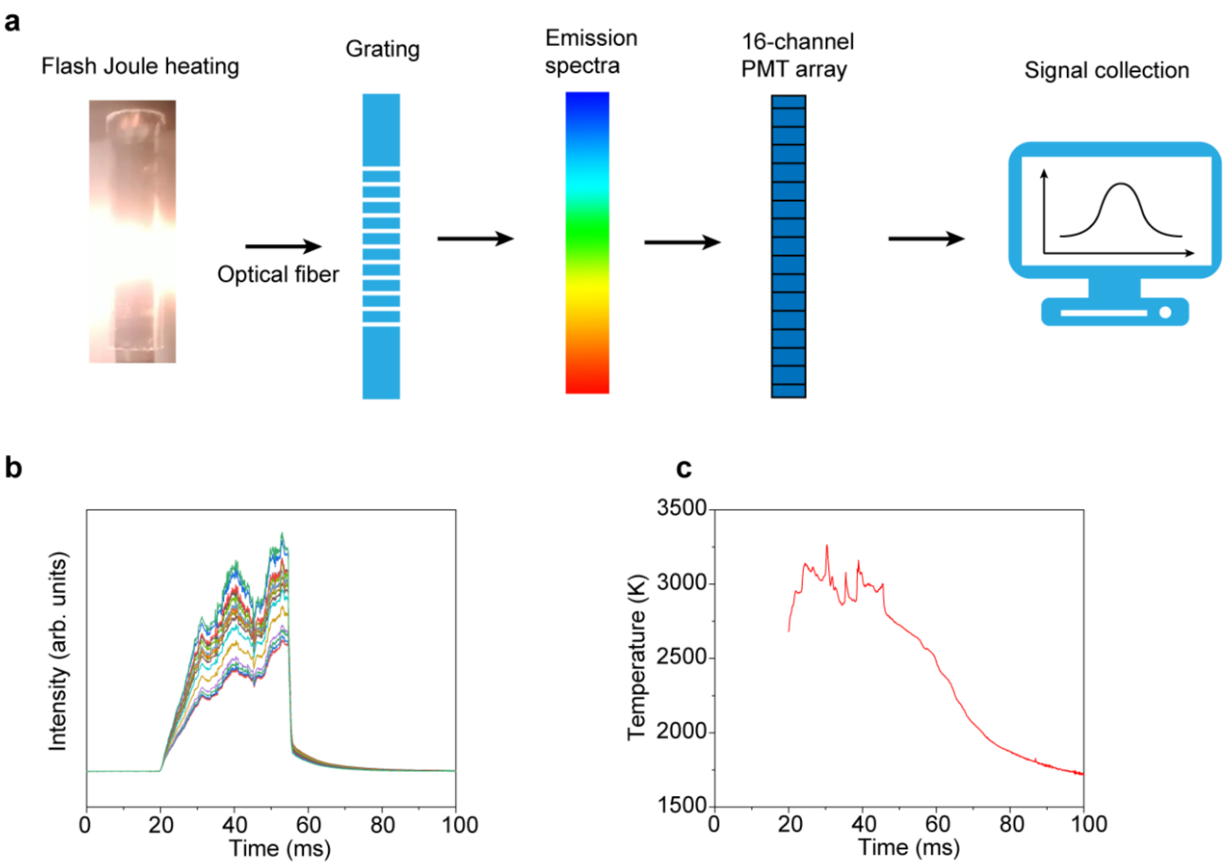

Supplementary Fig. 2. Temperature measurement by fitting the blackbody radiation. (**a**) Schematic of the spectrometer configuration. The light emitted from the sample during flash Joule heating (FJH) was collected by an optical fiber and separated by a grating box. The spectral radiance was recorded by a 16-channel photodiode array (Hamamatsu S4100-16R) at 640–1000 nm and transferred to digital signal, which was collected using a National Instrument multifunction I/O device PCIe-6320. (**b**) The normalized intensity of the recorded spectral radiance from 16 channels. (**c**) A representative real-time temperature curve by fitting the blackbody radiation. The spectra fitting was conducted using MATLAB[8].

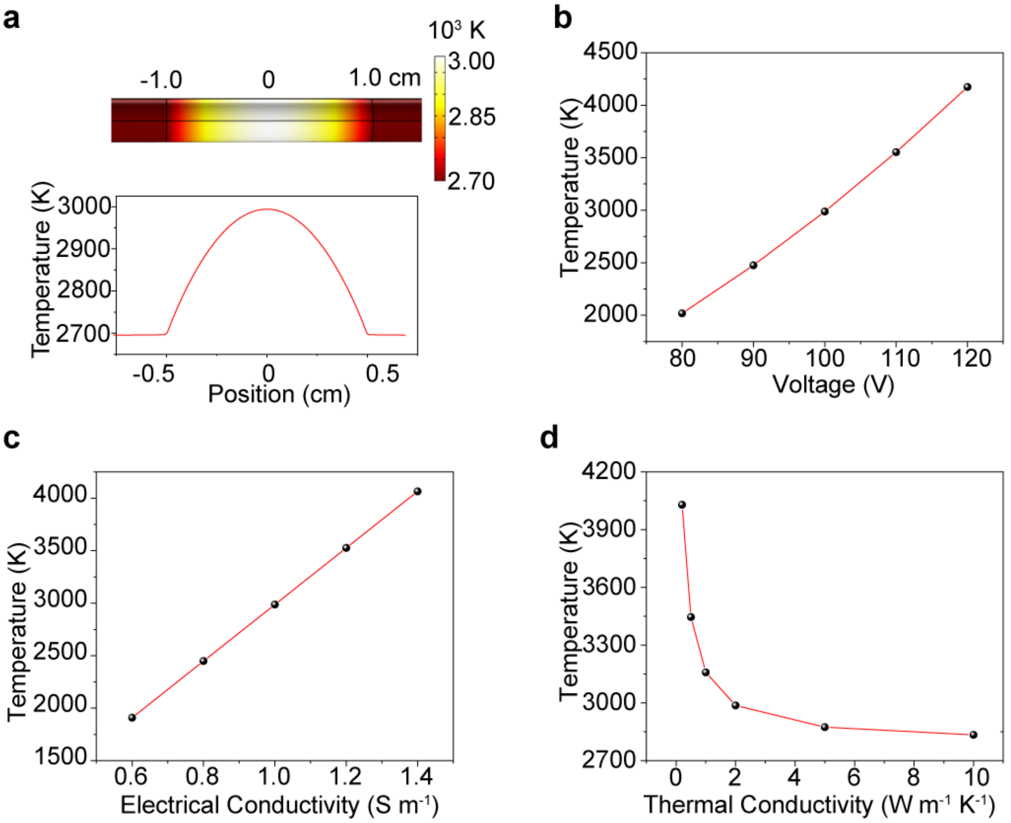

Supplementary Fig. 3. Simulation of temperature under different parameters. (**a**) Simulated temperature distribution of the sample during FJH (top), and the temperature profile (bottom). (**b**) Temperature varied with input voltage at a fixed electrical conductivity of 1 S m^{-1} and thermal conductivity of 1 W m^{-1} K^{-1}. (**c**) Temperature varied with electrical conductivity of the sample at a fixed voltage of 100 V and thermal conductivity of 1 W m^{-1} K^{-1}. (**d**) Temperature varied with thermal conductivity of the sample at a fixed voltage of 100 V and electrical conductivity of 1 S m^{-1}. The temperature is primarily affected by the flash Joule heating (FJH) voltage, and the materials properties, especially the electrical conductivity and thermal conductivity. The temperature is nearly linearly increased with FJH voltage and electrical conductivity of the materials.

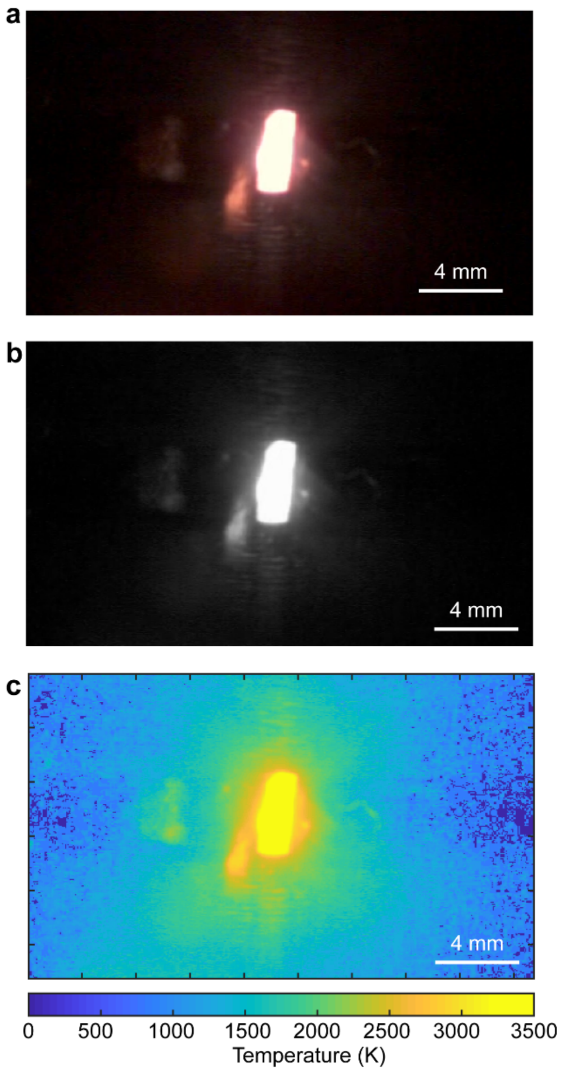

Supplementary Fig. 4. Temperature distribution within the sample during flash Joule heating (FJH). (**a**) Optical image of a sample recorded using an ultrafast camera during FJH. (**b**) The greyscale image. (**c**) The temperature distribution map.

According to the Stefan-Boltzmann law, the blackbody radiant emittance (j^*) is directly proportional to the fourth power of the blackbody's thermodynamic temperature (T),

$$j^* = \sigma T^4 \qquad (11)$$

where σ is a constant of proportionality. Hence, the temperature could be evaluated based on the radiant intensity. Experimentally, we recorded the optical image of a sample during FJH using an

ultrafast camera (**Supplementary Fig. 4a**). Then, the color image was converted to a grayscale image (**Supplementary Fig. 4b**), and then an intensity matrix using MATLAB. The highest temperature was measured by fitting the blackbody radiation (**Supplementary Fig. 2**), which corresponds to the largest value (I_{max}) in the intensity matrix. As an example, we used a FJH voltage of 120 V with the highest temperature (T_{max}) of ~3242 K. The temperature (T) of each pixel in the image was then calculated based on the Stefan-Boltzmann law using the intensity value (I) in the matrix,

$$\frac{I}{I_{max}} = (\frac{T}{T_{max}})^4 \qquad (12)$$

Then, the temperature distribution was plotted (**Supplementary Fig. 4c**). It is found that the temperature is very uniform throughout the whole sample.

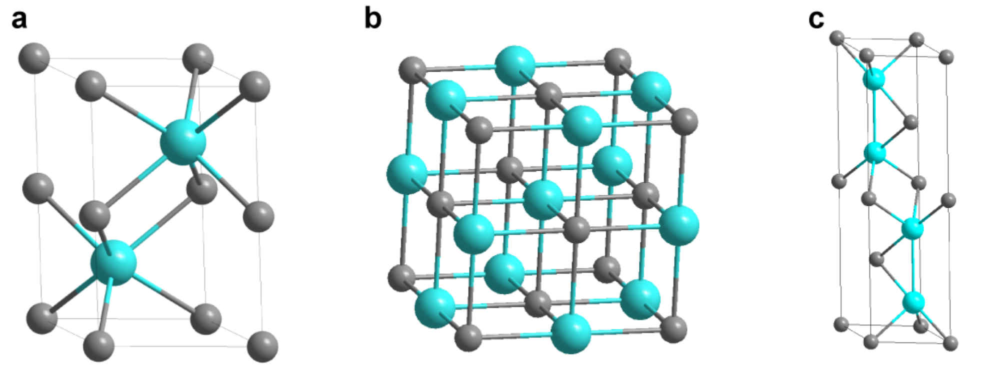

	β-Mo_2C	α-MoC_{1-x}	η-MoC_{1-x}
Structure	Hexagonal	Cubic	Hexagonal
Stacking sequence	ABAB	ABCABC	ABCABC
Space group	P63/mmc	Fm-3m	P63/mmc

Supplementary Fig. 5. Crystal structure, stacking sequence, and space group of three phases of molybdenum carbide. (**a**) β-Mo_2C. (**b**) α-MoC_{1-x}. (**c**) η-MoC_{1-x}.

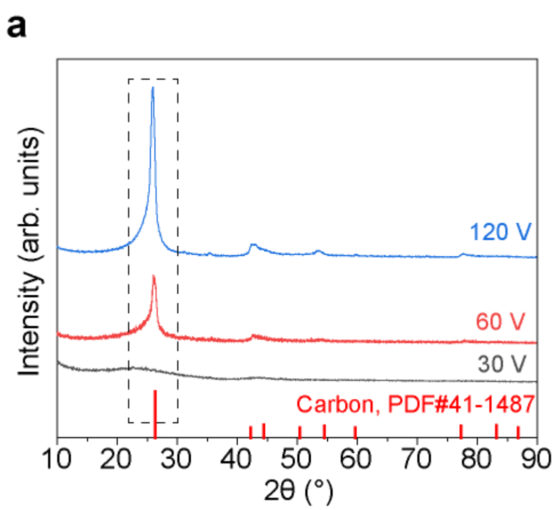

Supplementary Fig. 6. X-ray diffraction (XRD) of carbon black after flash Joule heating (FJH). (**a**) XRD patten of carbon black after FJH at different voltages. The graphite pattern (hexagonal, P63/mmc, PDF #41-1487) is shown as reference. (**b**) Zoom-in XRD pattern as shown in the black dash rectangle in **a**.

Amorphous carbon black was used as the conductive additive as well as carbon precursor for the carbothermic reduction. After FJH, the amorphous carbon black is graphitized, and the degree is related to the FJH voltages. A low FJH voltage (~30 V) cannot result in obvious graphitization (**Supplementary Fig. 6a**). As the voltage increases to 60 V, a sharp peak at ~26° is observed. The peak position is related to the FJH voltage and shifted downwards with regard to the (002) peak of graphite (**Supplementary Fig. 6b**). This material is termed as flash graphene (FG) as shown in our previous publication[7]. In **Fig. 2a**, **Figs. 5b-d**, and **Supplementary Figs. 23, 24, and 40**, the peak at ~26° are attributed to the graphene support.

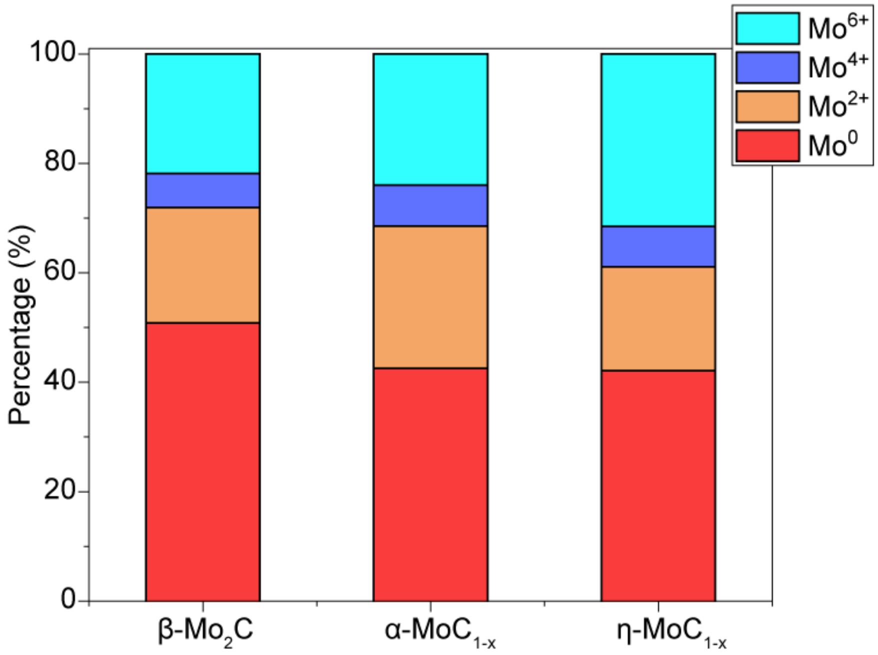

Supplementary Fig. 7. The content of Mo chemical states in three phases by X-ray photoemission spectroscopy (XPS). The Mo^0 and Mo^{2+} peaks are attributed to the molybdenum carbide due to the coexistence of Mo-Mo and Mo-C bonds in molybdenum carbides[9]. Mo^{4+} and Mo^{6+} are assigned to MoO_2 and MoO_3, respectively, due to the surface oxidation of molybdenum carbides when exposed to air[9,10]. The quantitative analysis of the ratios of four Mo chemical states shows that the high oxidation states (Mo^{6+}) in η-MoC_{1-x} is larger than those in β-Mo_2C and α-MoC_{1-x}, indicating that η-Mo_2C is the most oxidation susceptible phase.

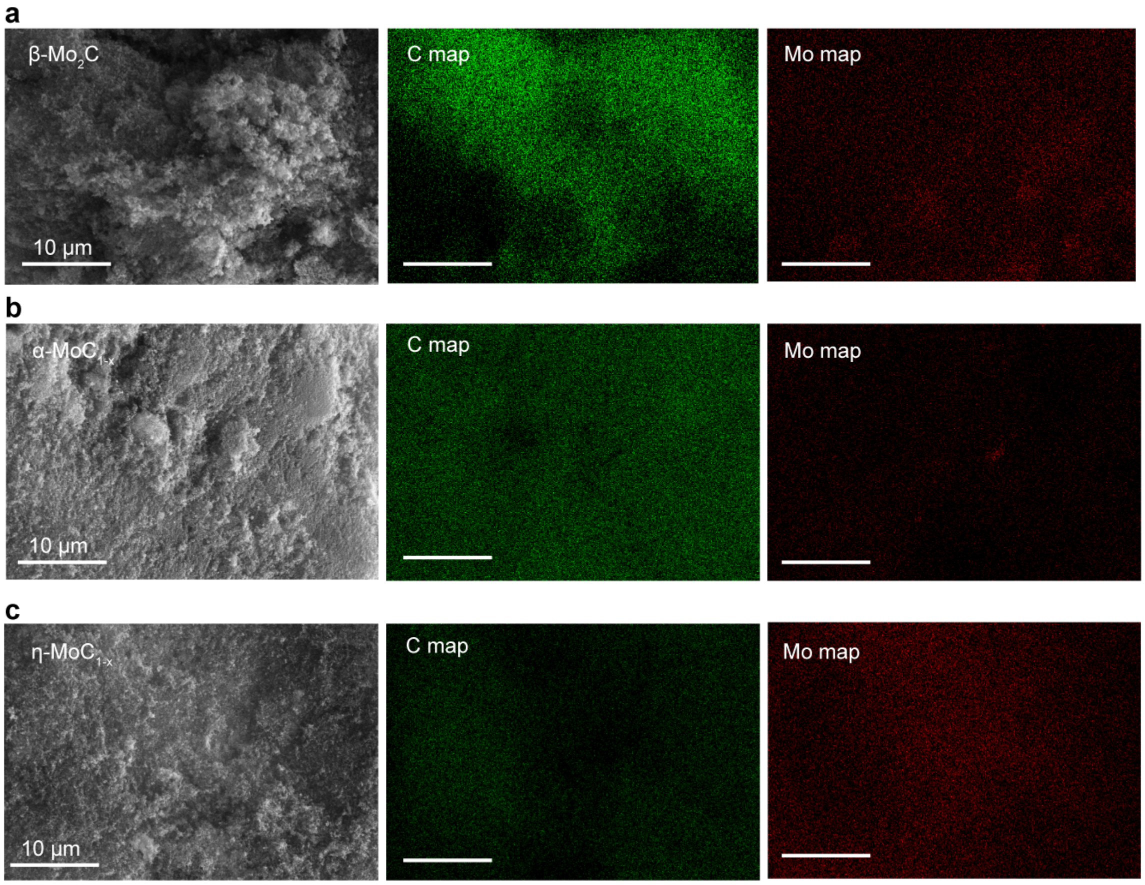

Supplementary Fig. 8. Scanning electron microscopy (SEM) images and energy dispersive spectroscopy (EDS) mapping of three phases of molybdenum carbide. (**a**) SEM image, C map, and Mo map of β-Mo_2C. (**b**) SEM image, C map, and Mo map of α-MoC_{1-x}. (**c**) SEM image, C map, and Mo map of η-MoC_{1-x}.

The SEM image shows the fine powder feature of all three carbide phases. The large-area EDS maps show uniform distribution of Mo and C, demonstrating that there is no large aggregation of carbide samples.

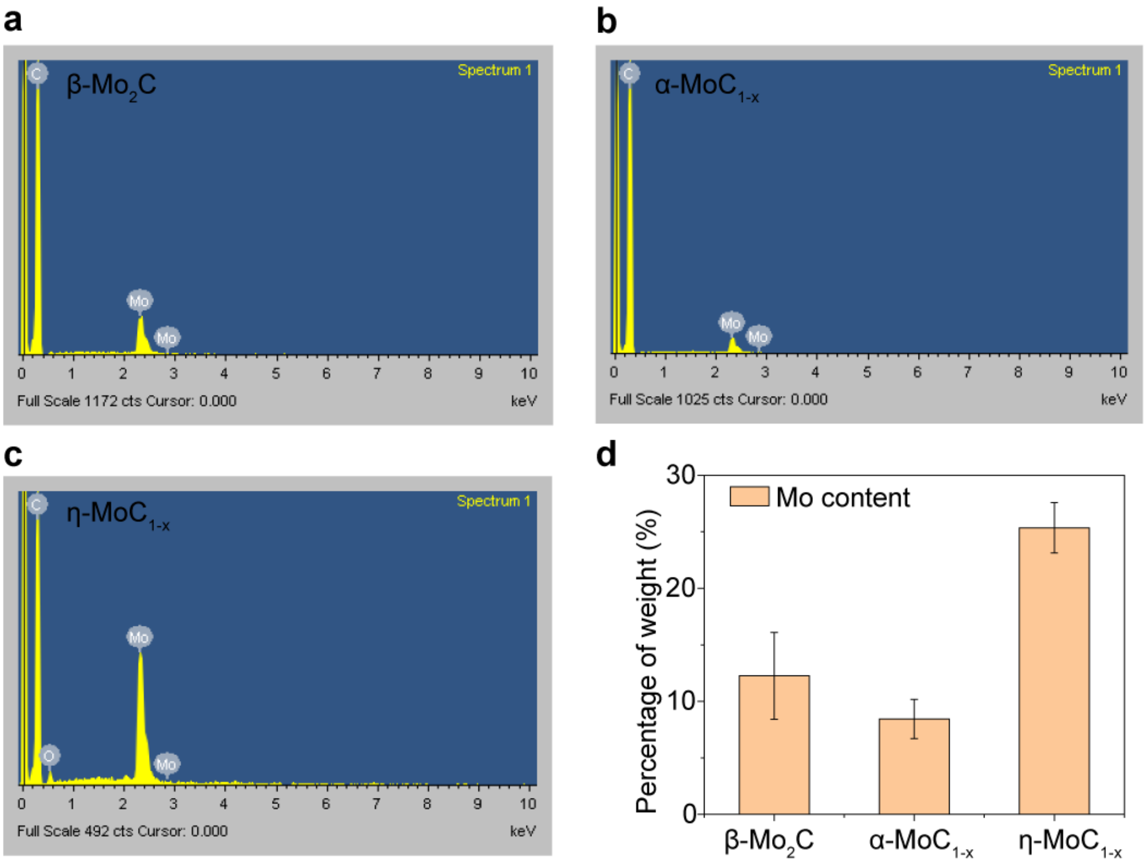

Supplementary Fig. 9. Elemental analysis of molybdenum carbides. (**a**) Element dispersive spectroscopy (EDS) spectrum of β-Mo_2C. (**b**) EDS spectrum of α-MoC_{1-x}. (**c**) EDS spectrum of η-MoC_{1-x}. (**d**) Mo content in three phases of molybdenum carbides. The scale bar denotes standard deviation where n = 3.

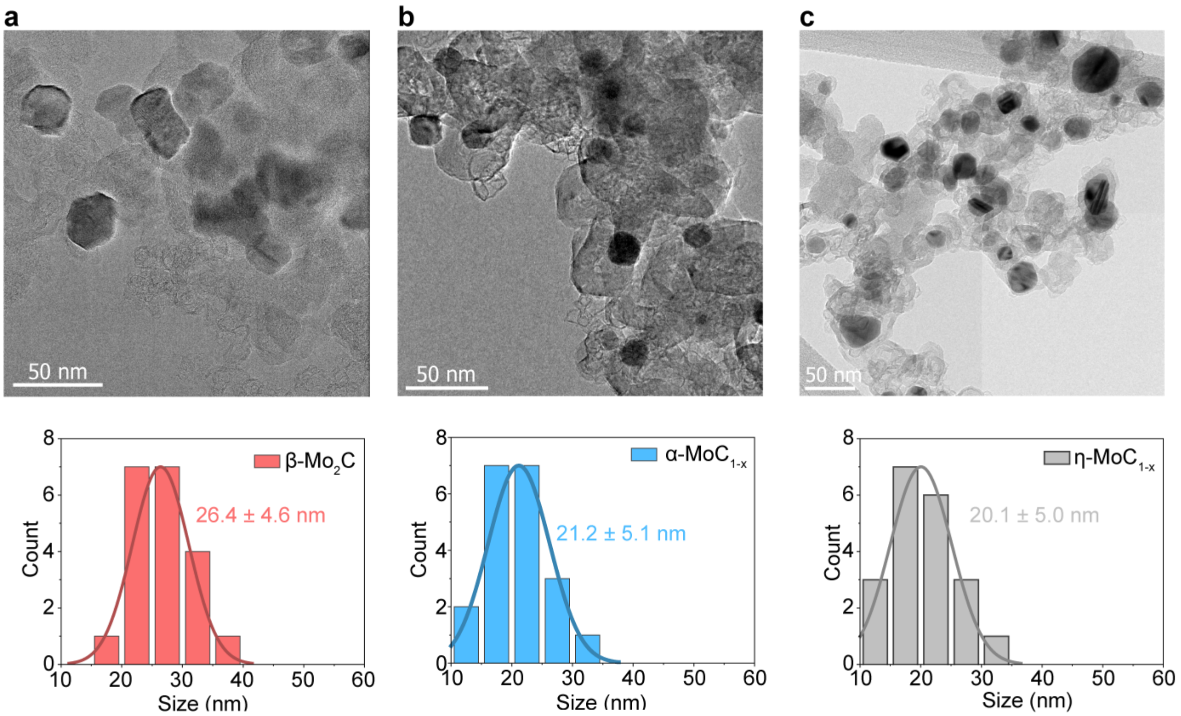

Supplementary Fig. 10. Size distribution of three phases of molybdenum carbide. (**a**) Bright-field transmission electron microscopy (BF-TEM) image of β-Mo_2C and the size distribution. (**b**) BF-TEM image of α-MoC_{1-x} and the size distribution. (**c**) BF-TEM image of η-MoC_{1-x} and the size distribution.

The particle sizes of the β-Mo_2C, α-MoC_{1-x}, and η-MoC_{1-x} are measured to be ~26.4 nm, ~21.2 nm, and ~20.1 nm, respectively, according to TEM statistics. Meanwhile, the crystal sizes of the β-Mo_2C, α-MoC_{1-x}, and η-MoC_{1-x} are determined to be ~22.3 nm, ~17.6 nm, and ~17.4 nm, respectively, according to XRD (**Supplementary Table 2**). The particle size values match well with the crystal size values, demonstrating that the as-synthesized particles are mostly single-crystal.

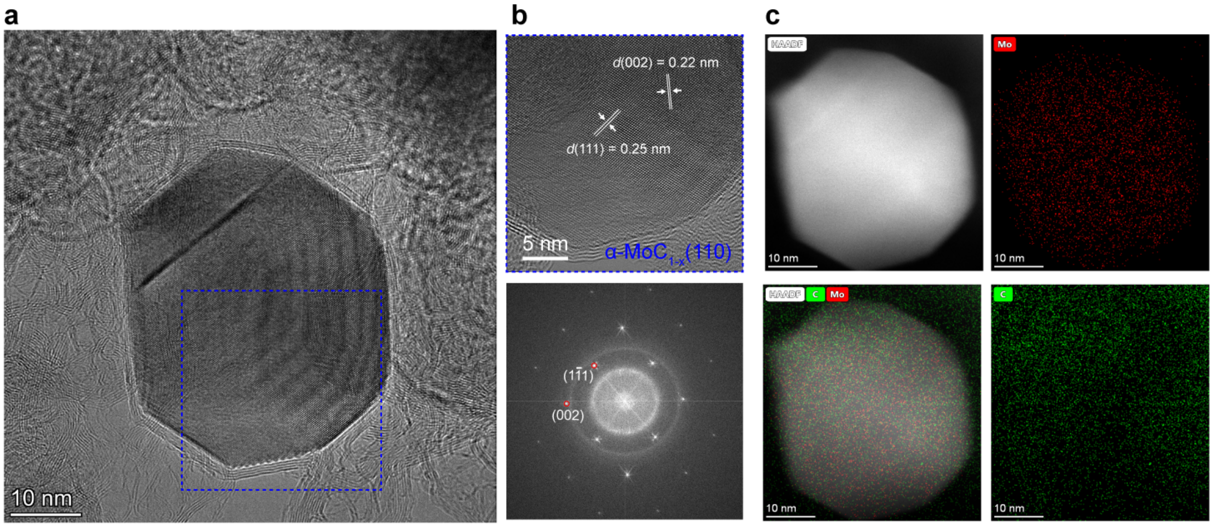

Supplementary Fig. 11. Transmission electron microcopy (TEM) characterization of α-MoC_{1-x} nanocrystals. (**a**) High-resolution transmission electron microscopy (HRTEM) image of an α-MoC_{1-x} nanoplate. (**b**) Zoom-in HRTEM of α-MoC_{1-x} (top) in the blue rectangle region in **a** and corresponding fast Fourier transform (FFT) pattern (bottom). (**c**) High-angle annular dark-field scanning transmission electron microscopy (HAADF-STEM) image of an α-MoC_{1-x} nanocrystal, and the energy dispersive spectroscopy (EDS) maps of Mo, C, and the overlapped image.

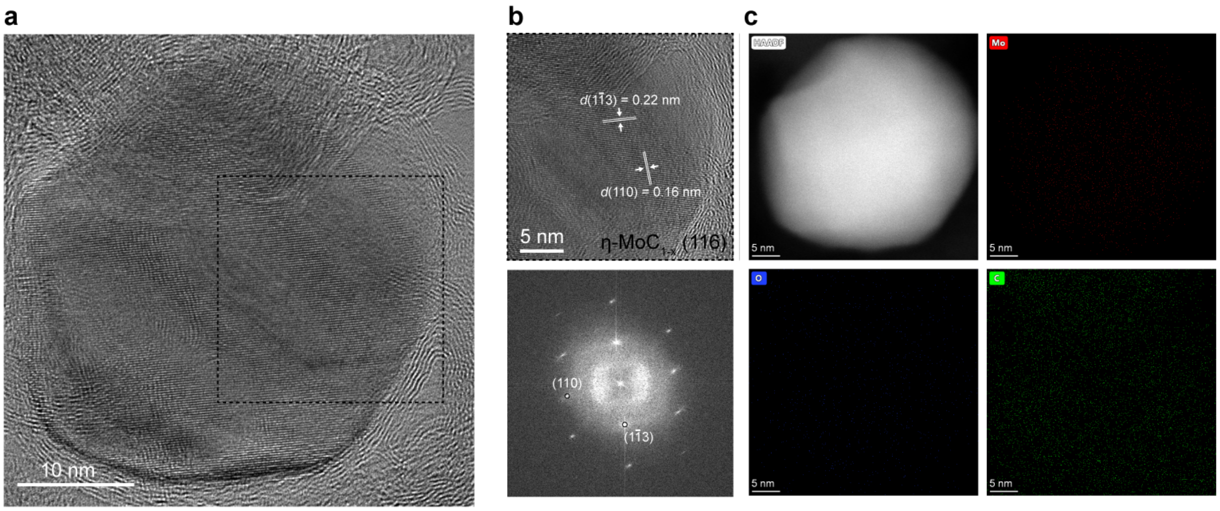

Supplementary Fig. 12. Transmission electron microscopy (TEM) characterization of η-MoC_{1-x} nanocrystals. (**a**) High-resolution transmission electron microscopy (HRTEM) image of an η-MoC_{1-x} nanocrystal. (**b**) Zoomed-in HRTEM of η-MoC_{1-x} (top) in the black rectangle region in **a** and corresponding fast Fourier transform (FFT) pattern (bottom). (**c**) High-angle annular dark-field scanning transmission electron microscopy (HAADF-STEM) image of an η-MoC_{1-x} nanocrystal, and the energy dispersive spectroscopy (EDS) maps of Mo, C, and O.

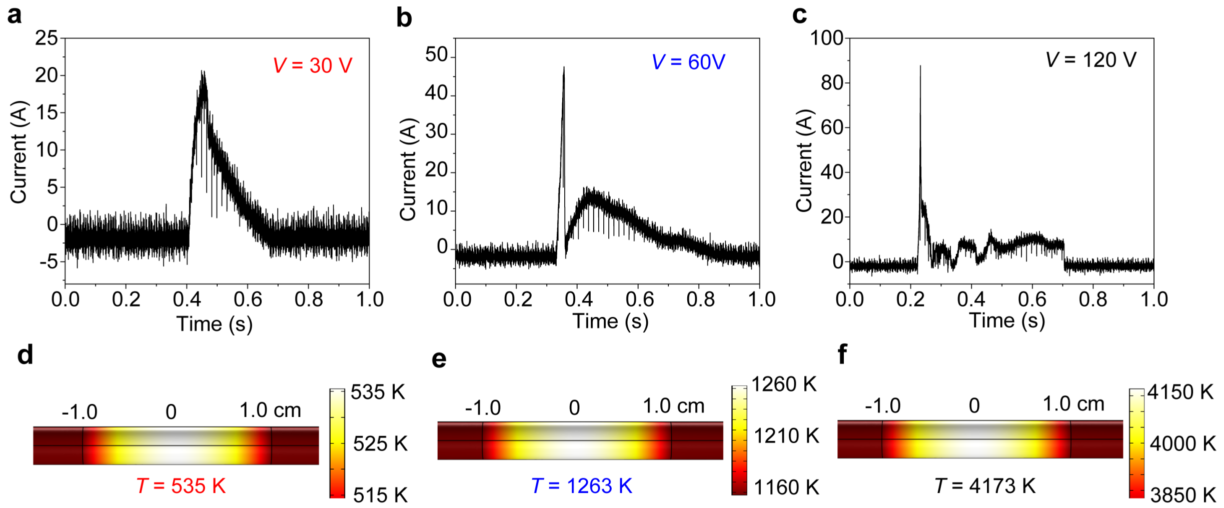

Supplementary Fig. 13. Current measurement and temperature simulation for flash Joule heating (FJH) at different voltages. (**a**) Current curve measured at FJH voltage (*V*) of 30 V. (**b**) Current curve measured at FJH voltage of 60 V. (**c**) Current curve measured at FJH voltage of 120 V. (**d**) Temperature simulation at FJH voltage of 30 V. (**e**) Temperature simulation at FJH voltage of 60 V. (**f**) Temperature simulation at FJH voltage of 120 V.

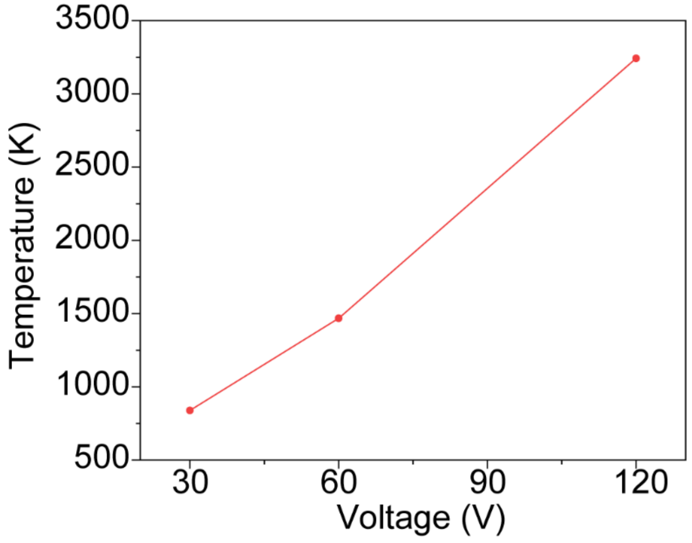

Supplementary Fig. 14. Maximum temperature measurement under different flash Joule heating (FJH) voltages.

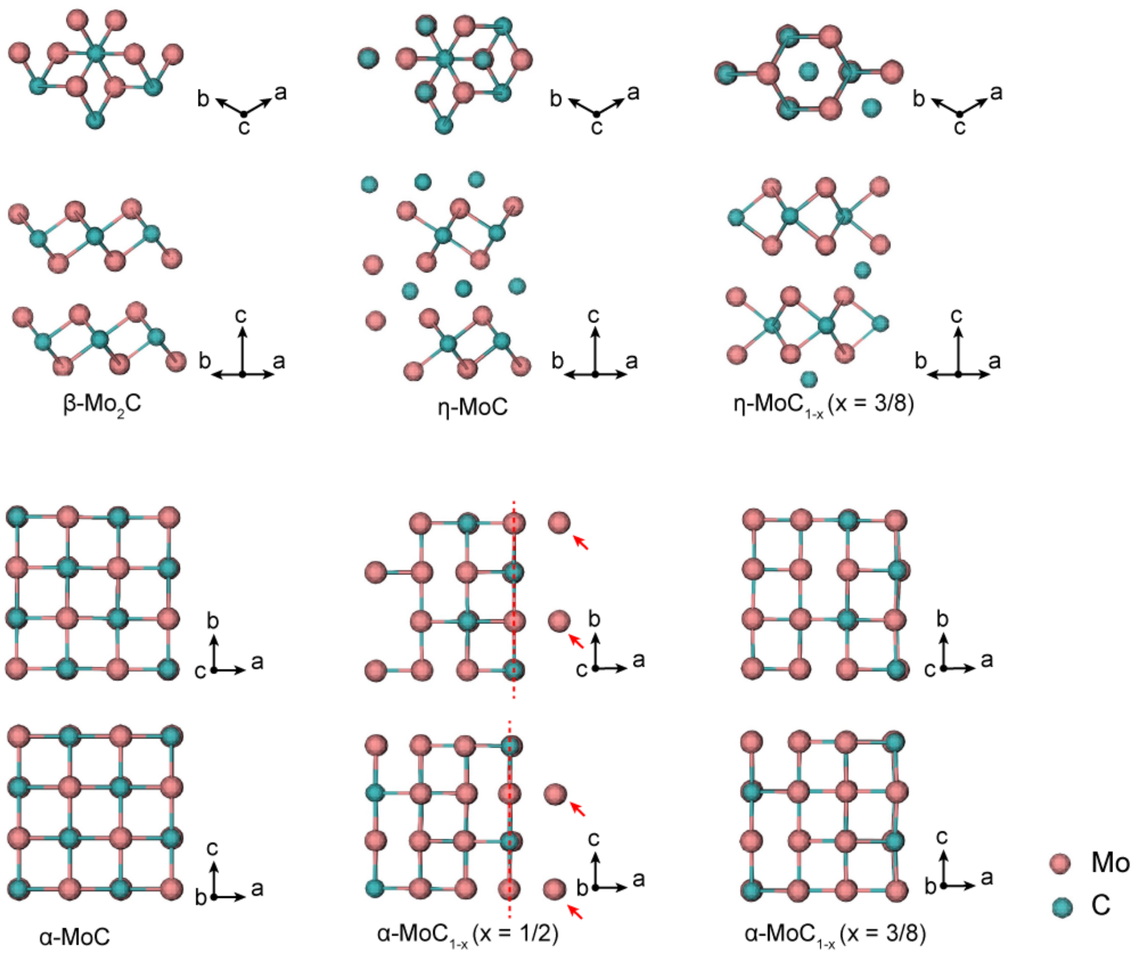

Supplementary Fig. 15. **Calculated crystal structures of β-Mo_2C, α-MoC_{1-x}, and η-MoC_{1-x} with different carbon contents.** For the structures with carbon vacancies, including α-MoC_{1-x} (x = 1/2), α-MoC_{1-x} (x = 3/8), and η-MoC_{1-x} (x = 3/8), the structures are the lowest-energy structures. The Mo atoms (red arrows) in the α-MoC_{1-x} (x = 1/2) structure are from the crystal plane (dash line) due to the lattice distortion induced by carbon vacancies.

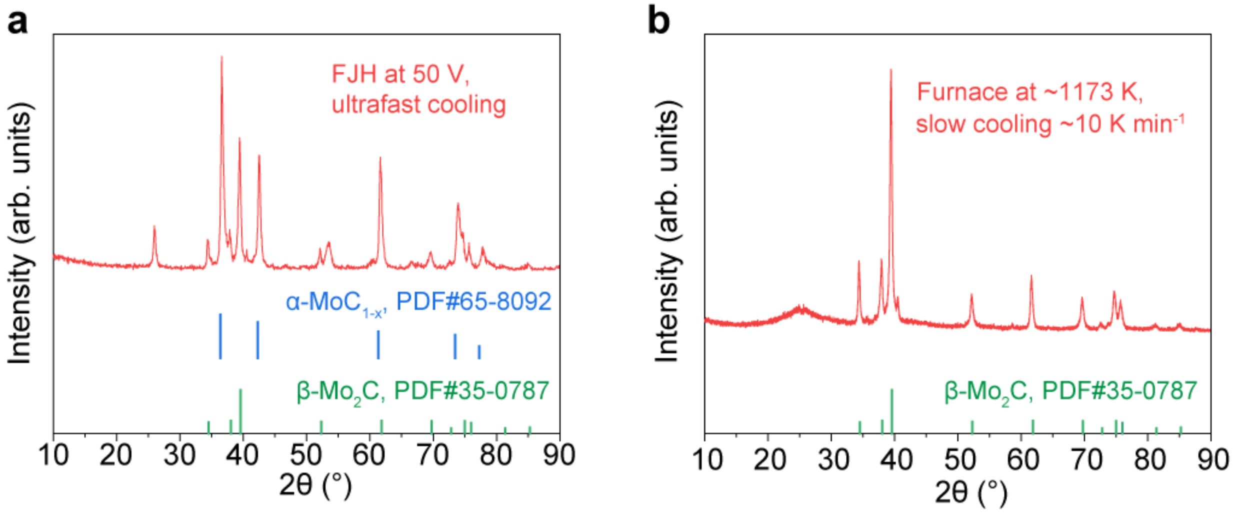

Supplementary Fig. 16. Cooling rate as the critical factor for metastable carbide phase synthesis. (**a**) X-ray diffraction (XRD) pattern of the samples synthesized using flash Joule heating (FJH) at voltage of 50 V and temperature of ~1173 K. (**b**) XRD pattern of the sample synthesized using tube furnace at 1173 K for 20 min and then cooling to room temperature at ~10 K min^{-1}.

By using a FJH voltage of 50 V, the metastable α-MoC_{1-x} phase was formed (**Supplementary Fig. 16a**). The maximum temperature at FJH voltage of 50 V was recorded to be ~1173 K. In contrast, when a tube furnace was used with a much slower cooling rate of ~10 K min^{-1} which could be considered as thermal equilibrium, under the same synthesis temperature, only the thermodynamically stable β-Mo_2C was synthesized (**Supplementary Fig. 16b**). This clearly demonstrated that the ultrafast cooling rate of the FJH process helps to kinetically retain the metastable carbide phases to room temperature.

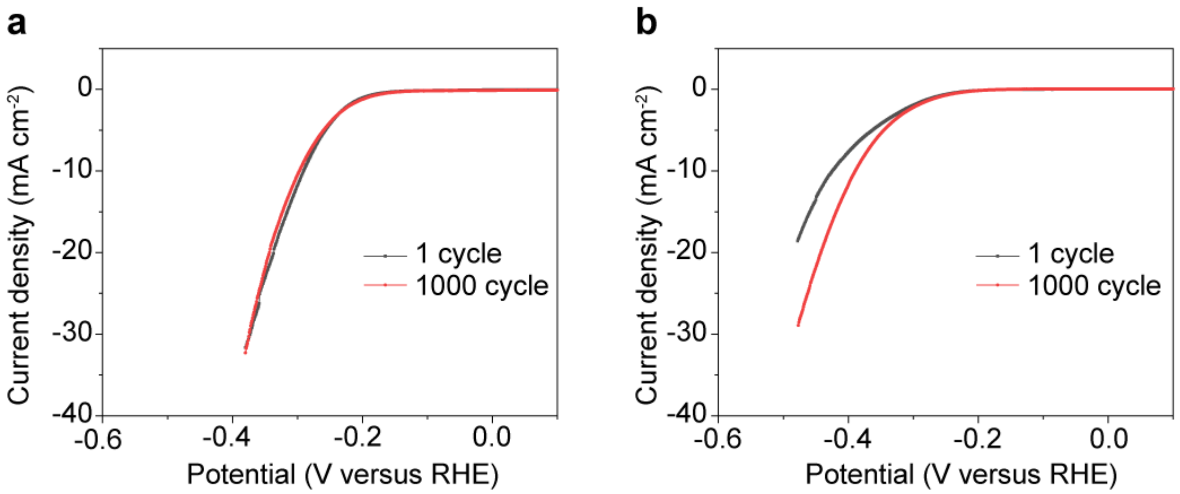

Supplementary Fig. 17. **Durability of molybdenum carbide electrocatalysts for hydrogen evolution reaction (HER) performance.** (**a**) Polarization curve of β-Mo_2C before and after 1000 potential cycles. (**b**) Polarization curve of η-MoC_{1-x} before and after 1000 potential cycles.

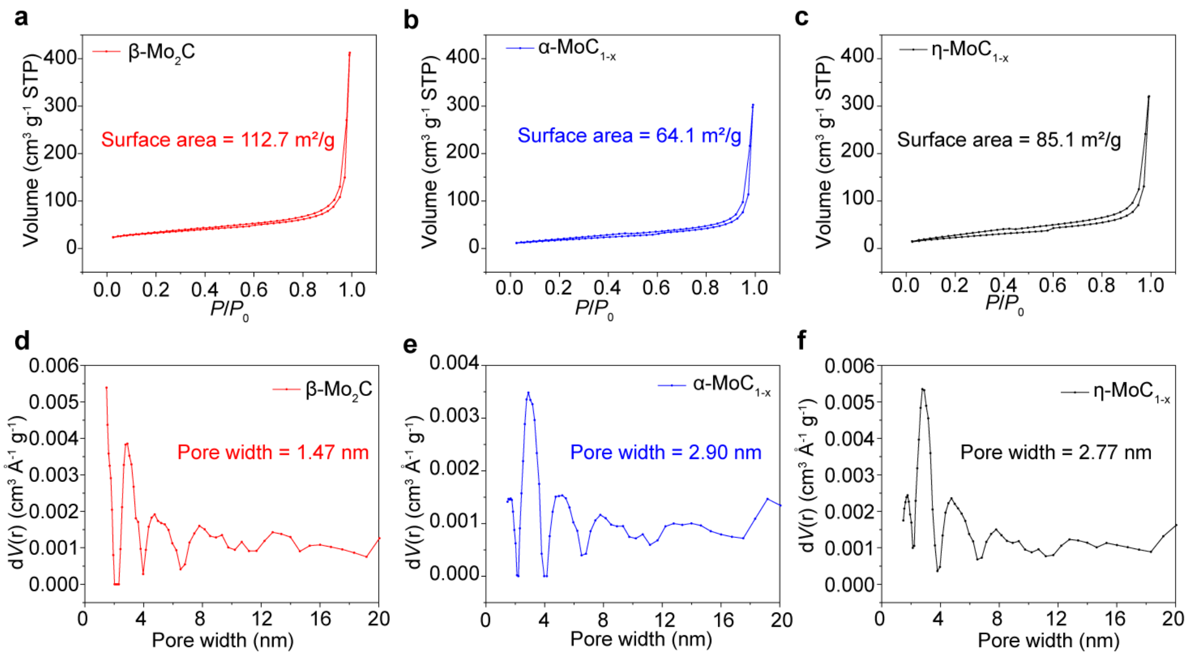

Supplementary Fig. 18. Brunauer–Emmett–Teller (BET) measurement of three phases of molybdenum carbides. (**a-c**) N_2 adsorption-desorption isotherms of β-Mo_2C (**a**), α-MoC_{1-x} (**b**), and η-MoC_{1-x} (**c**) at 77 K. (**d-f**) Pore width distribution of β-Mo_2C (**d**), α-MoC_{1-x} (**e**), and η-MoC_{1-x} (**f**) determined by the application of density functional theory (DFT) model. Note that the measured surface area and pore width are compositional results of molybdenum carbide nanocrystals and the graphene support.

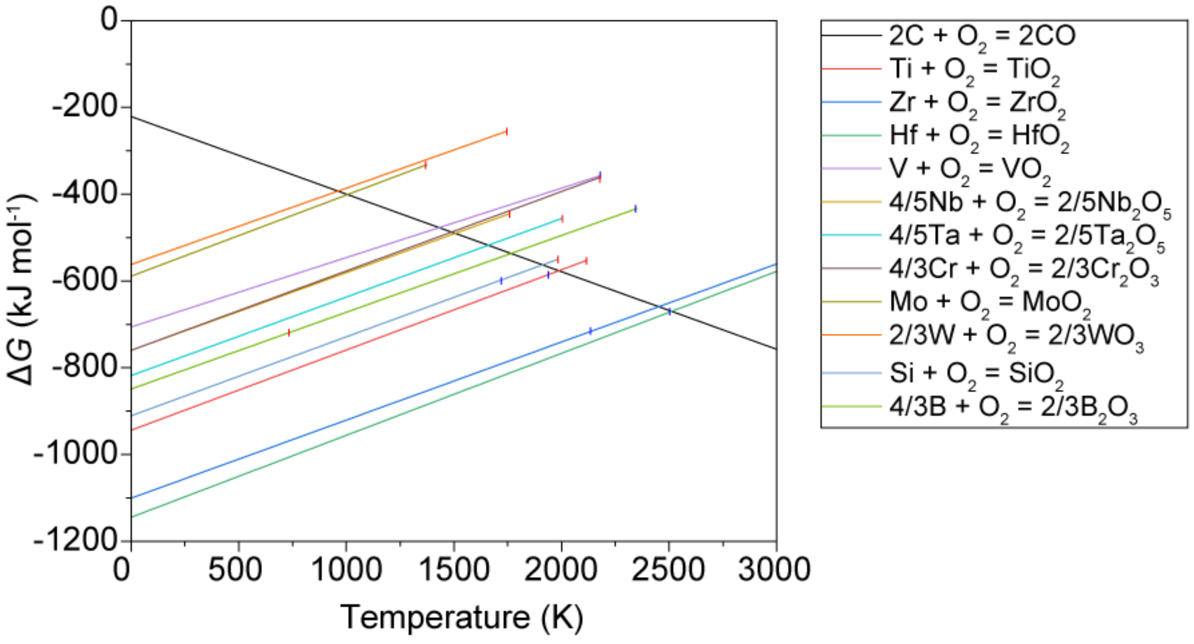

Supplementary Fig. 19. Ellingham diagram of CO and metal oxides. The green bars denote the melting points of the metal, and the red bars denote the melting points of the metal oxides.

According to the Ellingham diagram, the Gibbs free energy change (ΔG) curve of CO has negative slope, and the curves of the metal oxides have positive slopes. As a result, the metal oxides could be reduced to corresponding elemental metals as increasing the temperature. The cross points of CO curve and oxide curves represent the predicted reducing temperature.

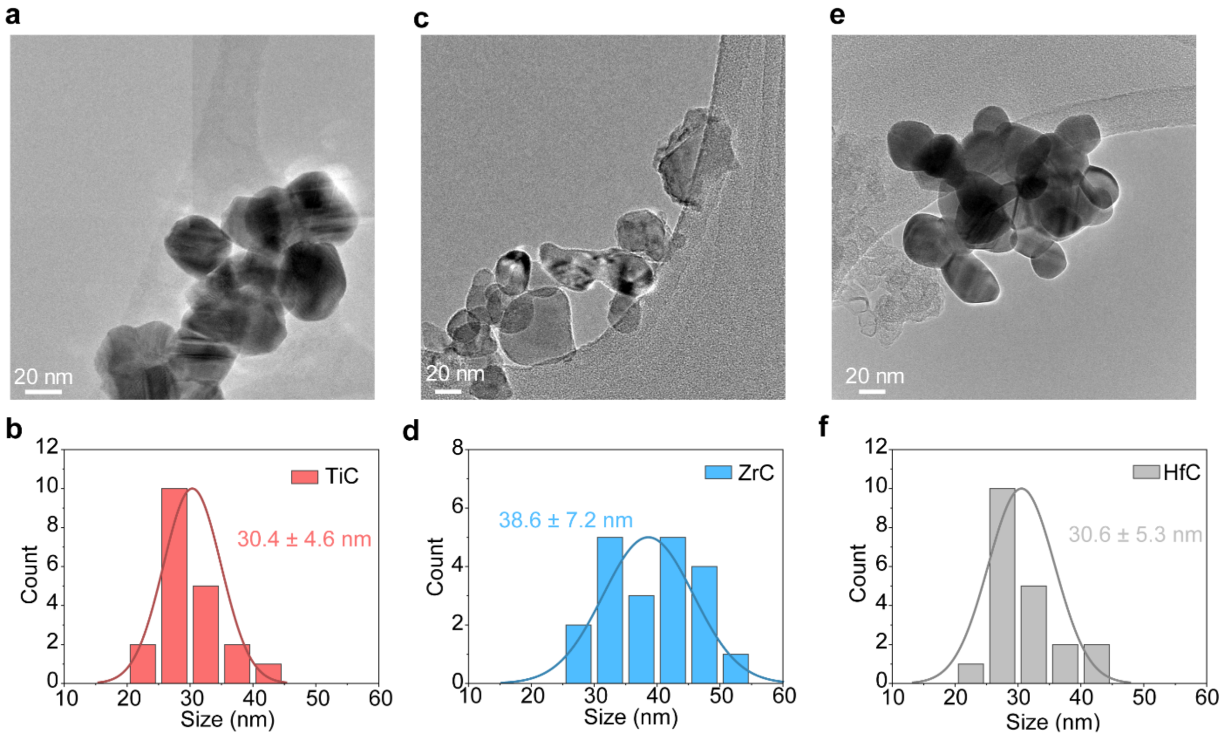

Supplementary Fig. 20. Size distribution of Group IVB metal carbides. (**a,b**) Bright-field transmission electron microscopy (BF-TEM) image of TiC (**a**) and the corresponding size distribution (**b**). (**c,d**) BF-TEM image of ZrC (**c**) and the corresponding size distribution (**d**). (**e,f**) BF-TEM image of HfC (**e**) and the corresponding size distribution (**f**).

The particle sizes of the TiC, ZrC, and HfC were measured to be ~30.4 nm, ~38.6 nm, and ~30.6 nm, respectively, according to TEM statistics. Meanwhile, the crystal sizes of the TiC, ZrC, and HfC were determined to be ~30.7 nm, ~40.6 nm, and ~30.8 nm, respectively, according to XRD (**Supplementary Table 2**). The particle size values match well with the crystal size values, demonstrating that the as-synthesized particles are mostly single-crystal.

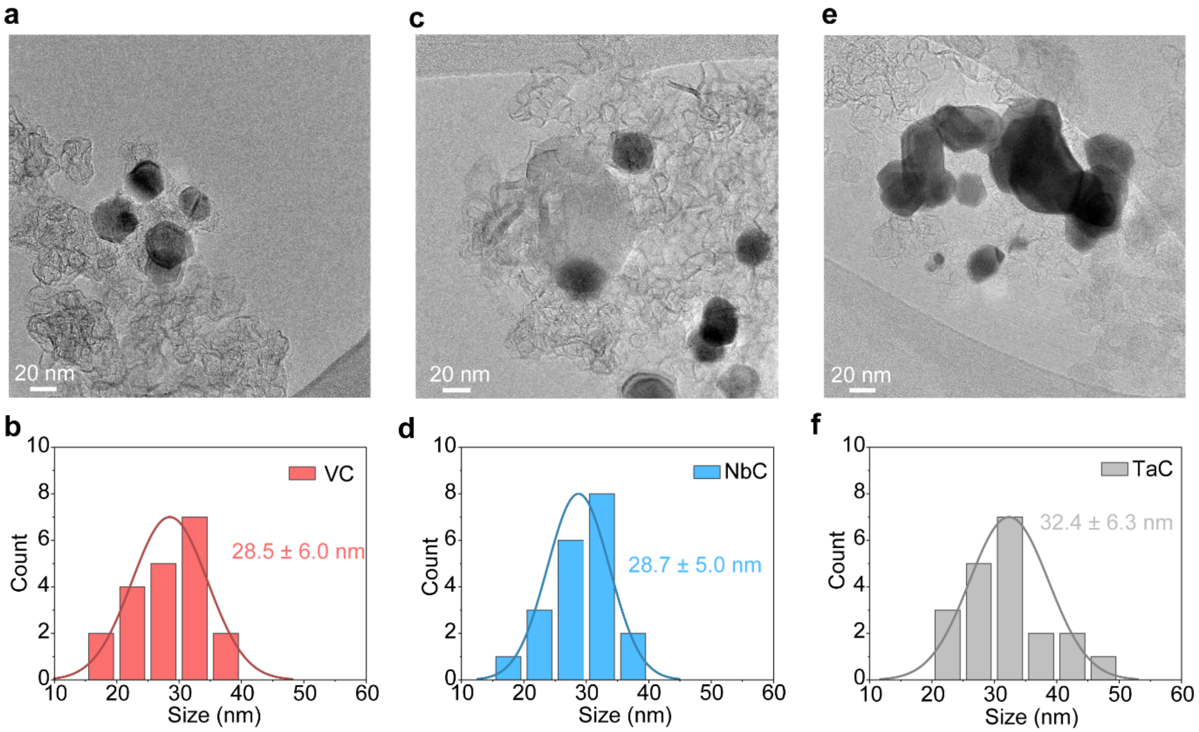

Supplementary Fig. 21. Size distribution of Group VB metal carbides. (**a,b**) Bright-field transmission electron microscopy (BF-TEM) image of VC (**a**) and the corresponding size distribution (**b**). (**c,d**) BF-TEM image of NbC (**c**) and the corresponding size distribution (**d**). (**e,f**) BF-TEM image of TaC (**e**) and the corresponding size distribution (**f**).

The particle sizes of the VC, NbC, and TaC nanocrystals were measured to be ~28.5 nm, ~28.7 nm, and ~32.4 nm, respectively, according to TEM statistics. Meanwhile, the crystal sizes of the VC, NbC, and TaC were determined to be ~26.6 nm, ~24.1 nm, and ~28.7 nm, respectively, according to XRD (**Supplementary Table 2**). The particle size values match well with the crystal size values, demonstrating that the as-synthesized particles are mostly single-crystal.

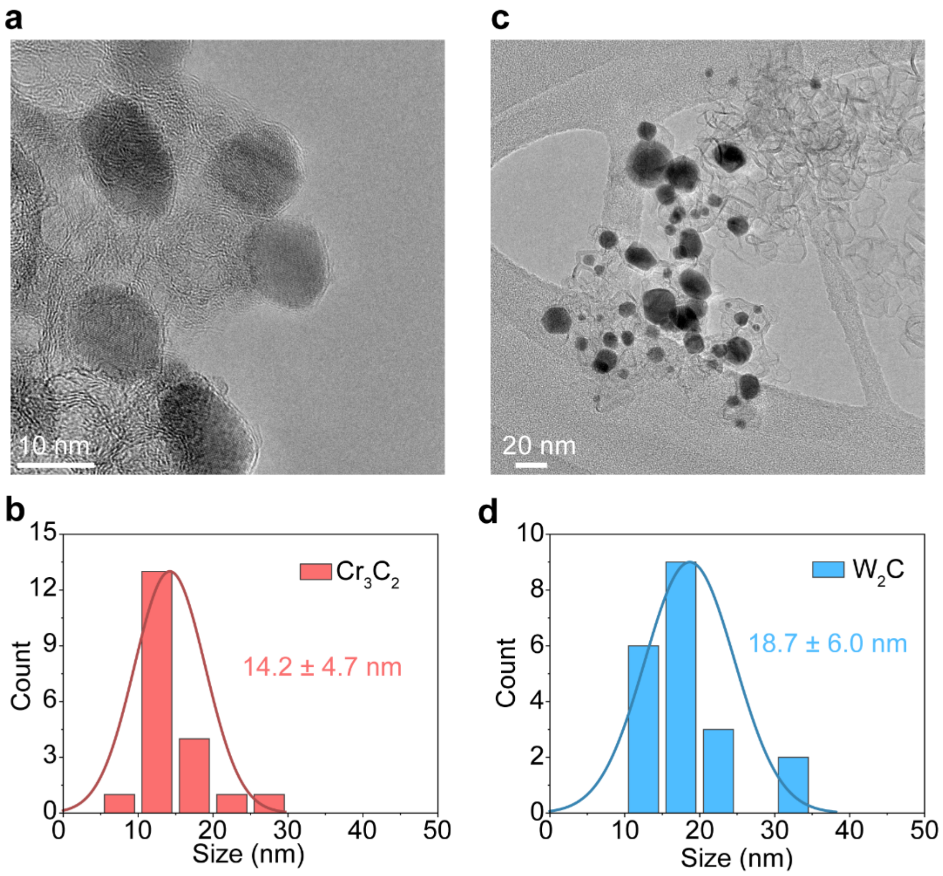

Supplementary Fig. 22. Size distribution of Group VIB metal carbides. (**a,b**) Bright-field transmission electron microscopy (BF-TEM) image (**a**) of Cr_3C_2 and the corresponding size distribution (**b**). (**c,d**) BF-TEM image of W_2C (**c**) and the corresponding size distribution (**d**).

The particle sizes of the Cr_3C_2 and W_2C were measured to be ~14.2 nm and ~18.7 nm, respectively, according to TEM statistics. Meanwhile, the crystal sizes of the Cr_3C_2 and W_2C were determined to be ~14.6 nm and ~25.1 nm, respectively, according to XRD (**Supplementary Table 2**). The particle size values match well with the crystal size values, demonstrating that the as-synthesized particles are mostly single-crystal.

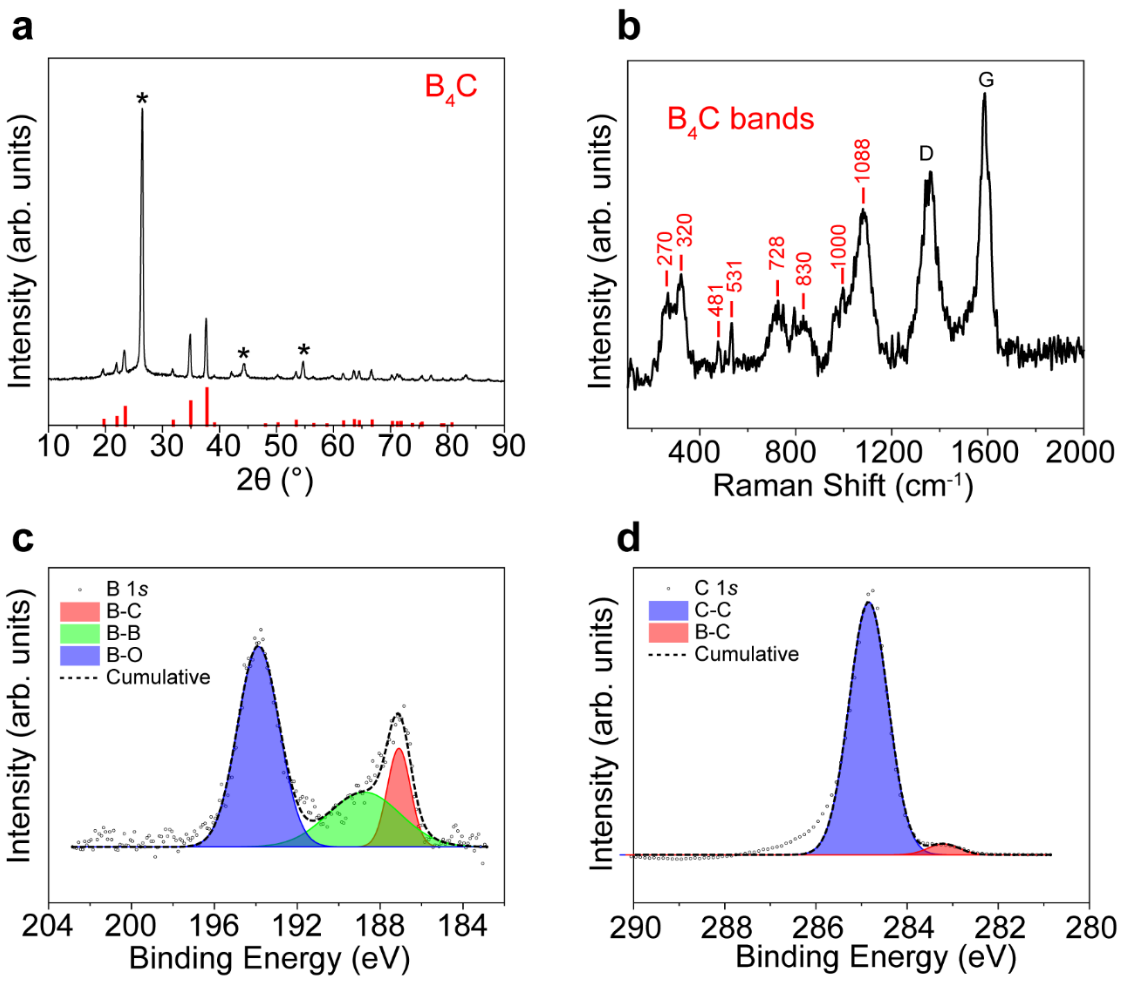

Supplementary Fig. 23. **Synthesis of boron carbide by flash Joule heating (FJH)**. (**a**) X-ray diffraction (XRD) pattern of B_4C. The PDF reference card is #35-0798. The stars indicate the peak from carbon support (PDF #41-1487). (**b**) Raman spectrum of B_4C. The Raman bands of B_4C is consistent with literature[11]. The Raman D band and G band of graphene support are also shown. (**c**) X-ray photoemission spectroscopy (XPS) fine spectrum of B 1*s*, demonstrating the existence of B-C. (**d**) XPS fine spectra of C 1*s*.

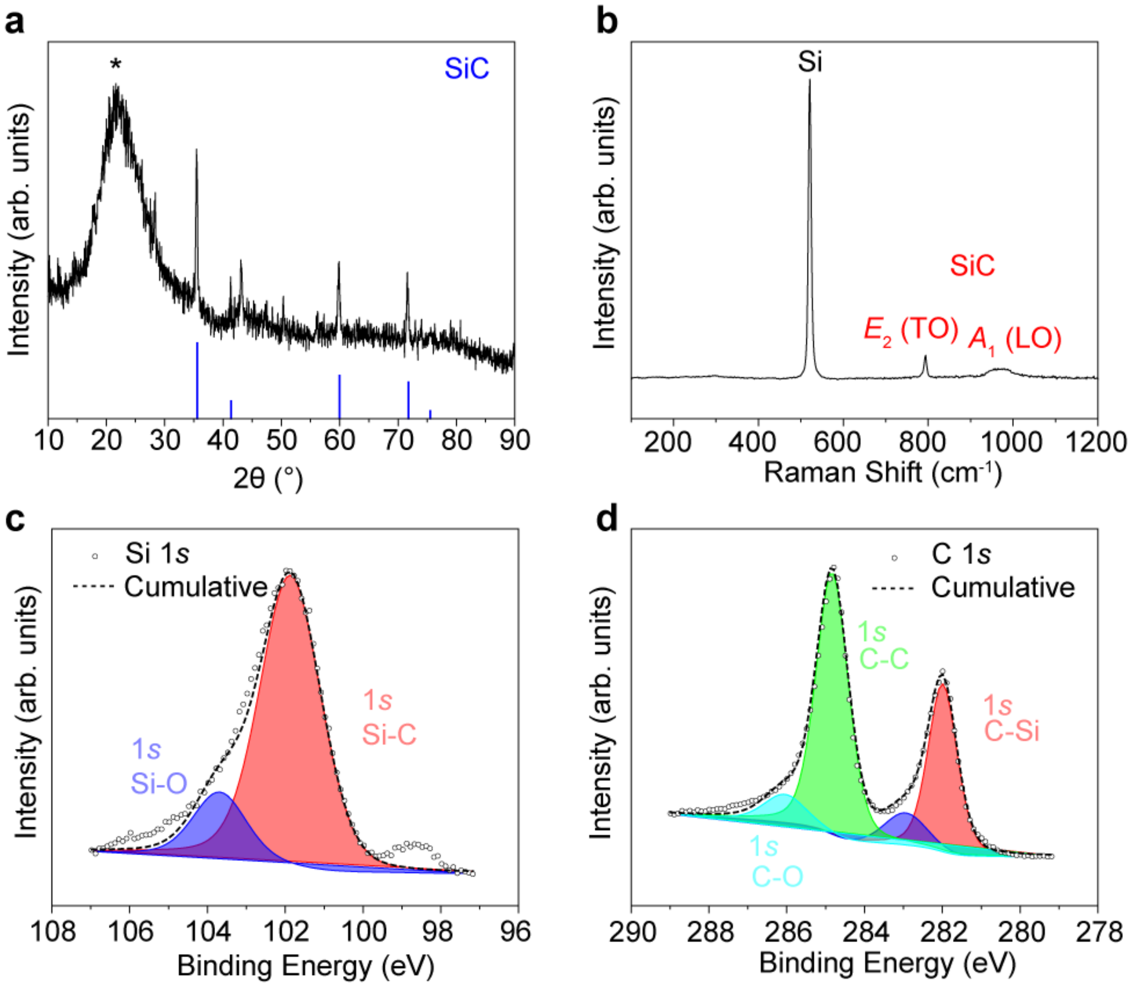

Supplementary Fig. 24. Synthesis of silicon carbide by flash Joule heating (FJH). (**a**) X-ray diffraction (XRD) pattern of SiC synthesized using the precursor of SiO_2 and carbon black with the mass ratio of 1 by 1. The broad peak at ~26° (star) is attributed to the carbon support. The PDF reference card for SiC is #29-1129. (**b**) Raman spectrum of SiC. The LO and TO modes of SiC are consistent with previous report[12]. The Raman band of Si is also identified, which could be the Si(0) residues reduced from SiO_2 during the carbothermic reduction process. (**c**) X-ray photoemission spectroscopy (XPS) fine spectrum of Si 1*s*. (**d**) XPS fine spectrum of C 1*s*. The XPS fine spectra proves the existence of Si-C.

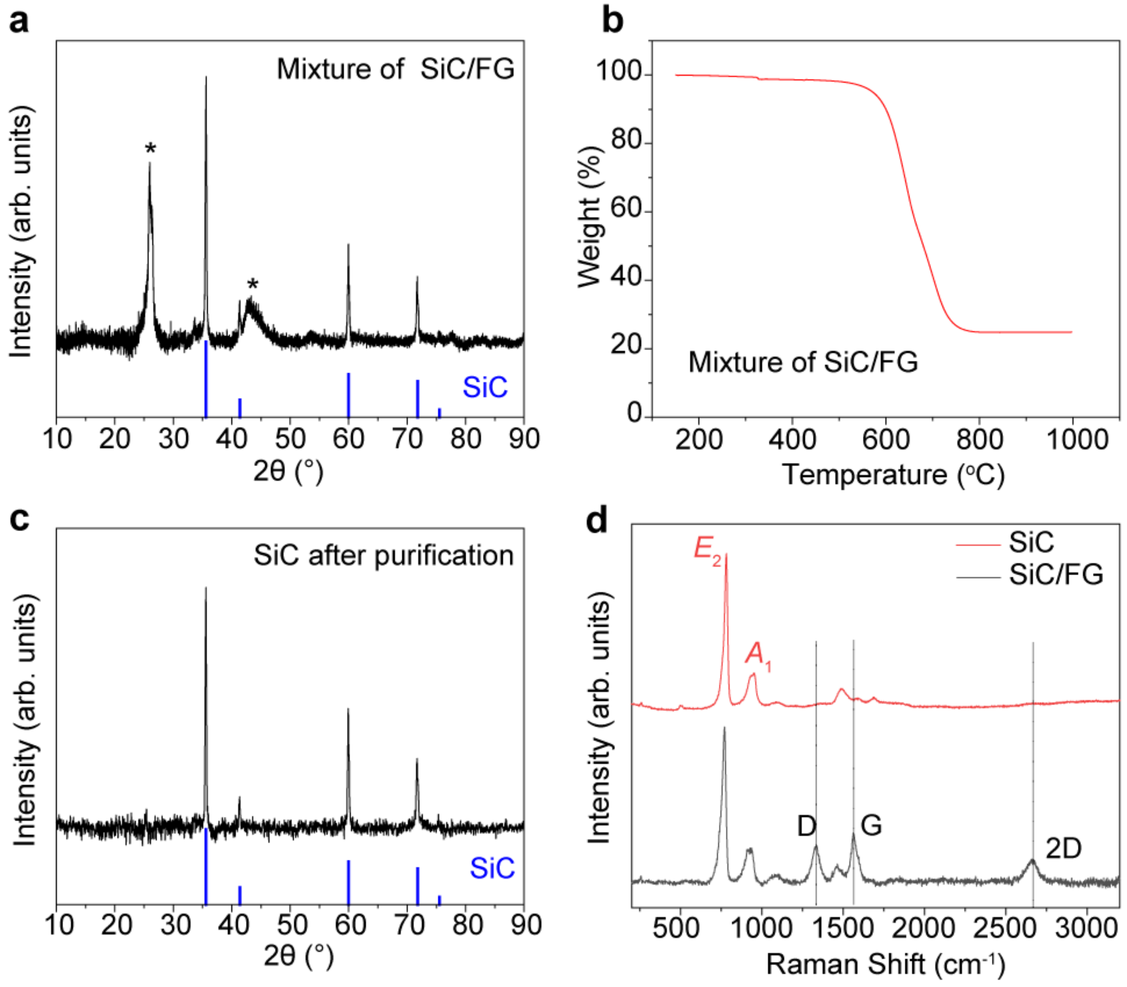

Supplementary Fig. 25. Purification of SiC. (**a**) XRD pattern of the as-synthesized mixture of SiC and flash graphene (FG). The reference PDF card for SiC is #29-1129. The stars denote the peaks from the graphene support. (**b**) TGA curve of the mixture of SiC/graphene in air at the heating rate of 10 °C min^{-1}. (**c**) XRD pattern of the purified SiC. The reference PDF card for SiC is #29-1129. (**d**) Raman spectra of the as-synthesized SiC/graphene mixture and the purified SiC.

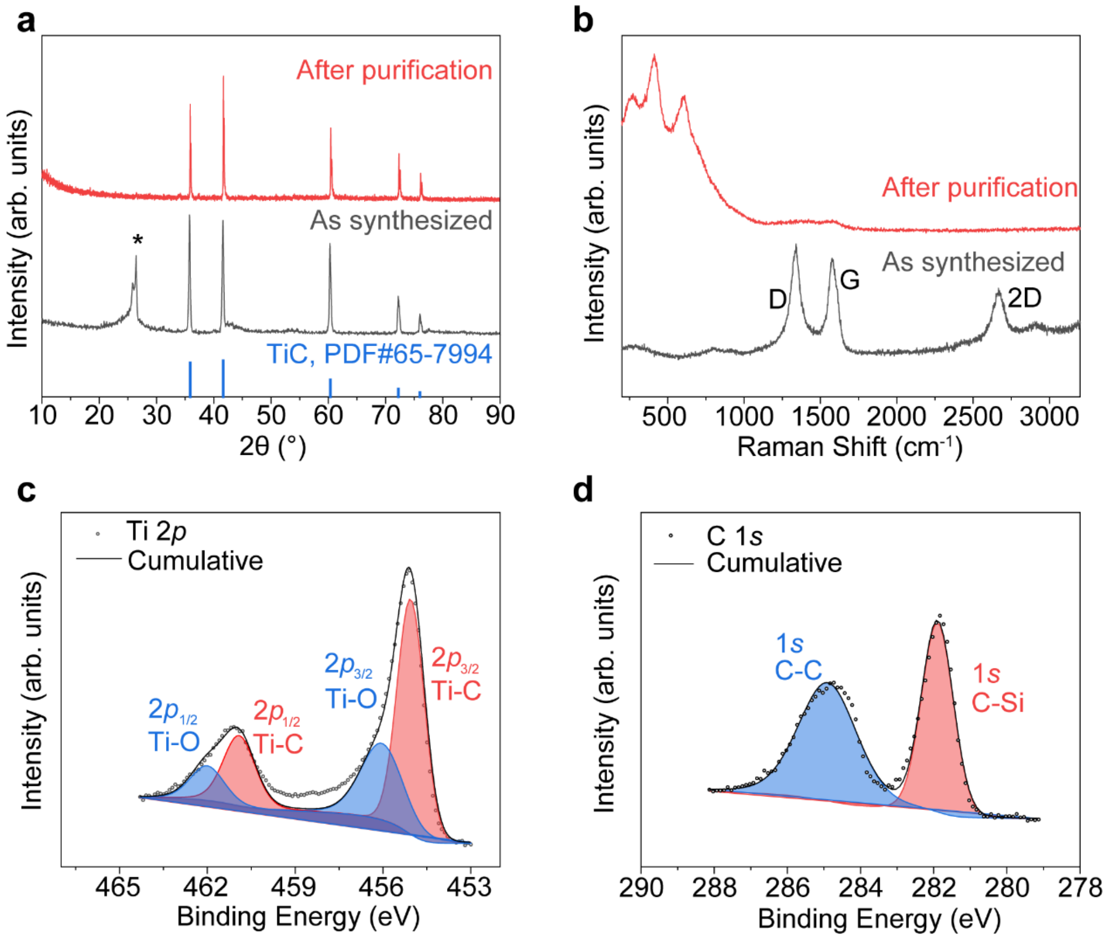

Supplementary Fig. 26. Purification of TiC nanocrystals. (**a**) XRD patterns of the as-synthesized mixture of TiC/graphene, and the TiC product after purification. The star denotes the peak from the graphene support. (**b**) Raman spectra of the as-synthesized mixture of TiC/graphene, and the TiC product after purification. (**c**) XPS fine spectrum of Ti of the purified TiC product. (**d**) XPS fine spectrum of C of the purified TiC product.

After purification, no graphene peak was identified in the XRD pattern, and no graphene band was identified in the Raman analysis, demonstrating the efficient removal of graphene. The XPS confirms the chemical states of Ti and C in TiC. The Ti-O could be due to the surface oxidation, and the C-C peak could be from the absorbed carbon in air.

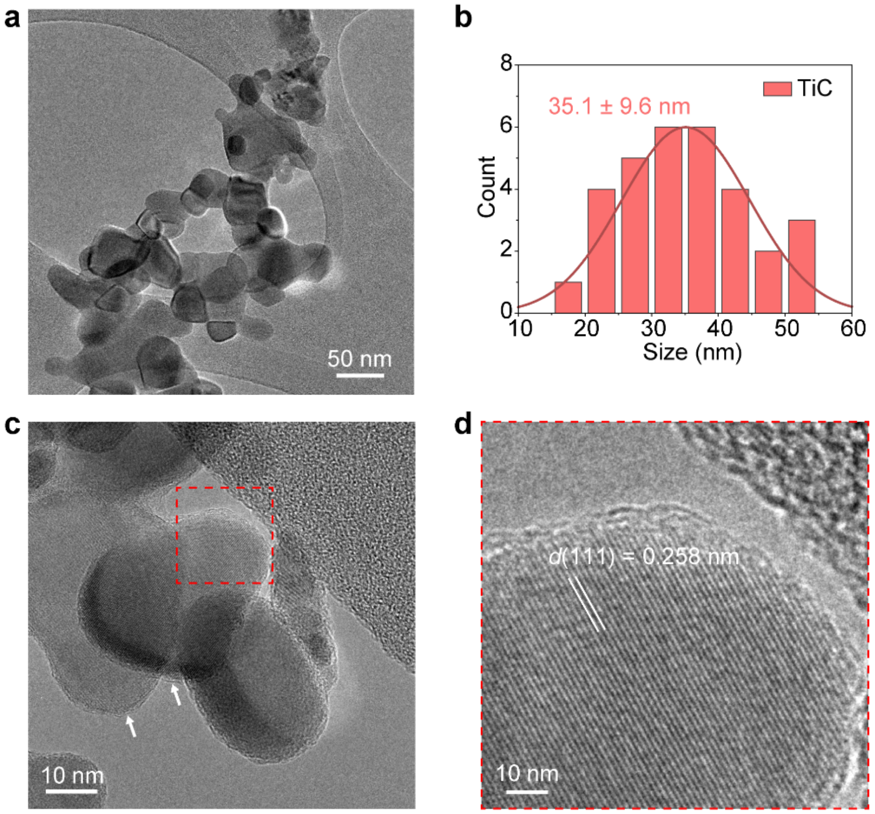

Supplementary Fig. 27. Transmission electron microscopy (TEM) characterization of TiC nanocrystals after purification. (**a**) Bright-field TEM (BF-TEM) image of TiC. (**b**) Particle size distribution of the TiC nanocrystals. (**c**) High-resolution TEM (HRTEM) image of the TiC nanocrystals. (**d**) Enlarged HRTEM image in the dashed rectangle in **c**. The interplanar spacing of 0.258 nm corresponds to the *d*(111) of TiC.

The low-magnification TEM image shows only the TiC nanoparticles without excess carbon. After purification, the particle size is measured to be ~35.1 nm, somewhat larger than that of the as-synthesized sample of ~30.4 nm, demonstrating that the purification process did not result in significant coarsening of the particles. The HRTEM image shows the clean surface of the particle without coverage of the carbon layer, showing the efficient removal of graphene.

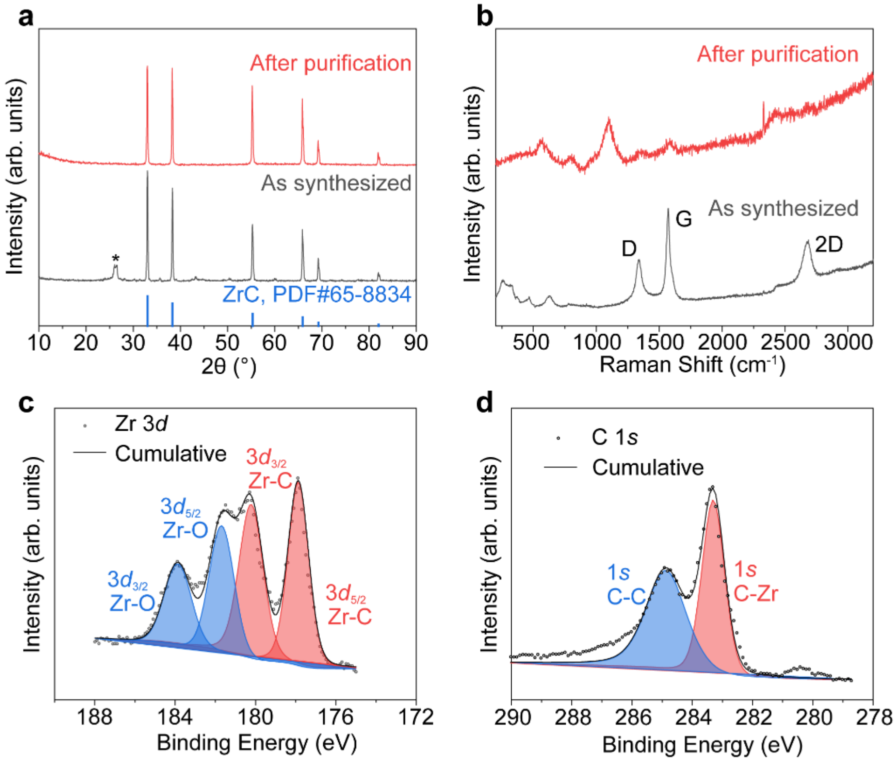

Supplementary Fig. 28. Purification of ZrC nanocrystals. (**a**) X-ray diffraction (XRD) patterns of the as-synthesized mixture of ZrC/graphene, and the ZrC product after purification. The star denotes the peak from the graphene support. (**b**) Raman spectra of the as-synthesized mixture of ZrC/graphene, and the ZrC product after purification. (**c**) X-ray photoemission spectroscopy (XPS) fine spectrum of Zr of the purified ZrC product. (**d**) XPS fine spectrum of C of the purified ZrC product.

After purification, no graphene peak was identified in the XRD pattern, and no graphene band was detected in the Raman spectrum, demonstrating the efficient removal of graphene. The XPS confirms the chemical states of Zr and C in ZrC. The Zr-O could be due to the surface oxidation, and the C-C peak could be from the absorbed carbon in air.

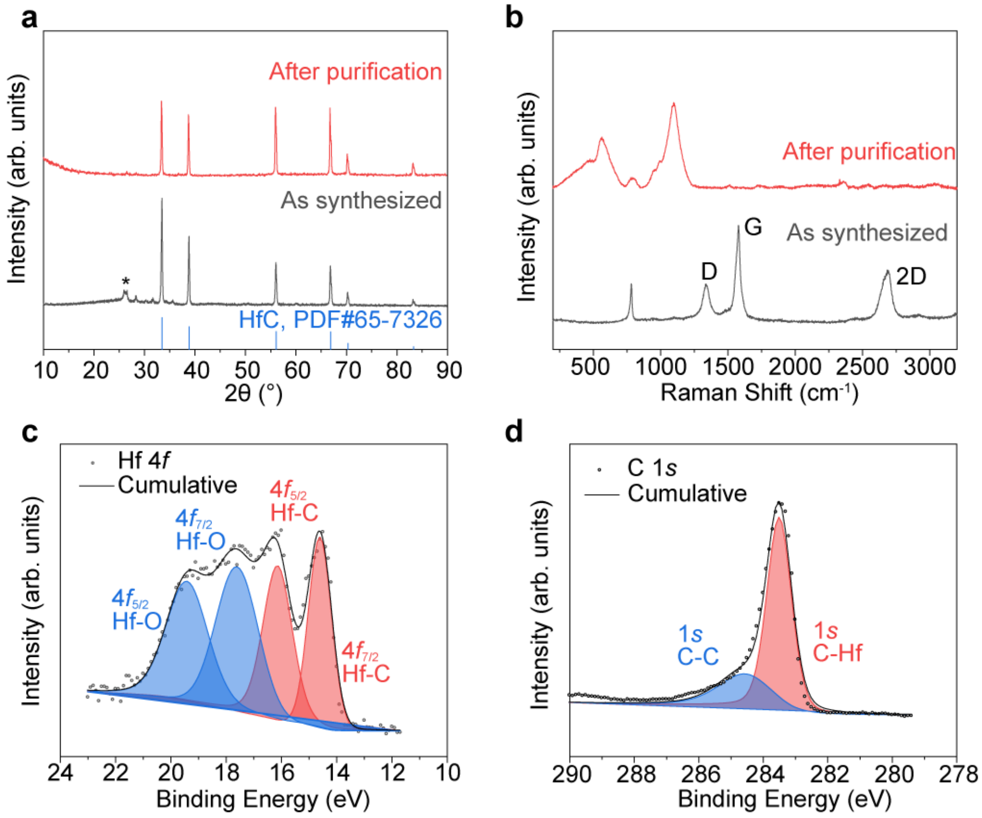

Supplementary Fig. 29. Purification of HfC nanocrystals. (**a**) X-ray diffraction (XRD) patterns of the as-synthesized mixture of HfC/graphene, and the HfC product after purification. The star denotes the peak from the graphene support. (**b**) Raman spectra of the as-synthesized mixture of HfC/graphene, and the HfC product after purification. (**c**) X-ray photoemission spectroscopy (XPS) fine spectrum of Hf in the purified HfC product. (**d**) XPS fine spectrum of C in the purified HfC product.

After purification, no graphene peak was identified in the XRD pattern, and no graphene band was detected in the Raman spectrum, demonstrating the efficient removal of graphene. The XPS confirms the chemical states of Hf and C in HfC. The Hf-O could be due to the surface oxidation, and the C-C peak could be from the absorbed carbon in air.

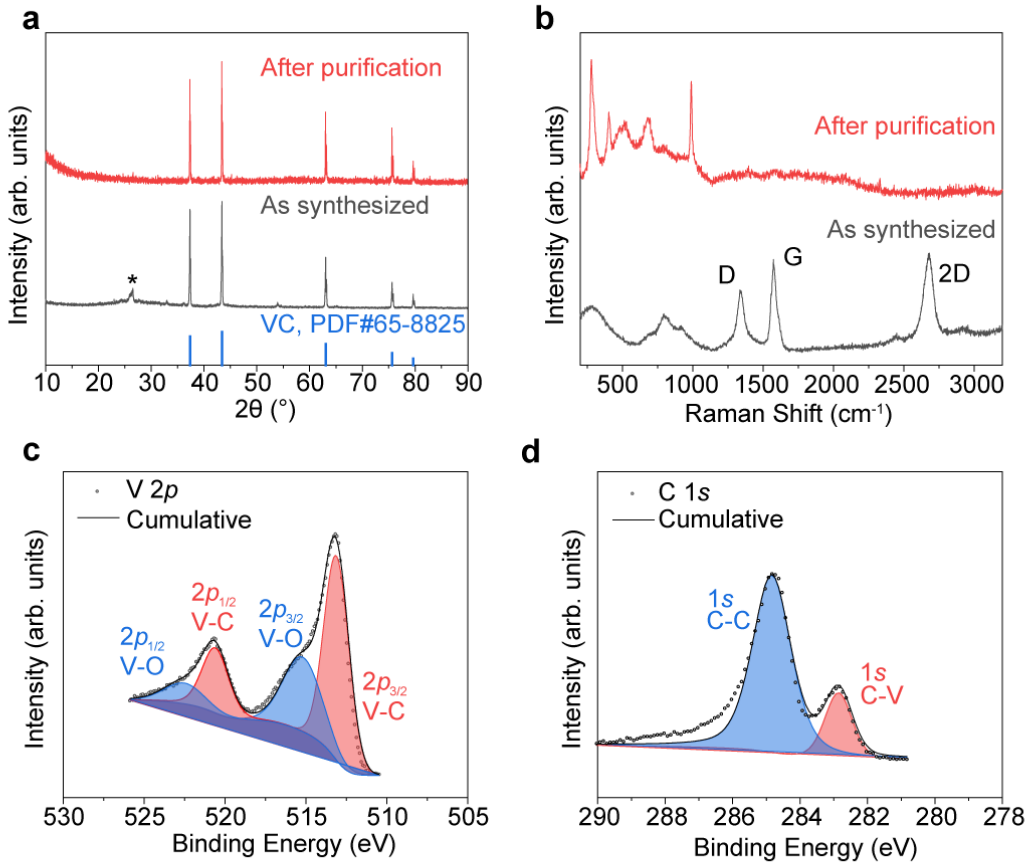

Supplementary Fig. 30. Purification of VC nanocrystals. (**a**) X-ray diffraction (XRD) patterns of the as-synthesized mixture of VC/graphene, and the VC product after purification. The star denotes the peak from the graphene support. (**b**) Raman spectra of the as-synthesized mixture of VC/graphene, and the VC product after purification. (**c**) X-ray photoemission spectroscopy (XPS) fine spectrum of V in the purified VC product. (**d**) XPS fine spectrum of C in the purified VC product.

After purification, no graphene peak was identified in the XRD pattern, and no graphene band was detected in the Raman spectrum, demonstrating the efficient removal of graphene. The XPS confirms the chemical states of V and C in VC. The V-O could be due to the surface oxidation, and the C-C peak could be from the absorbed carbon in air.

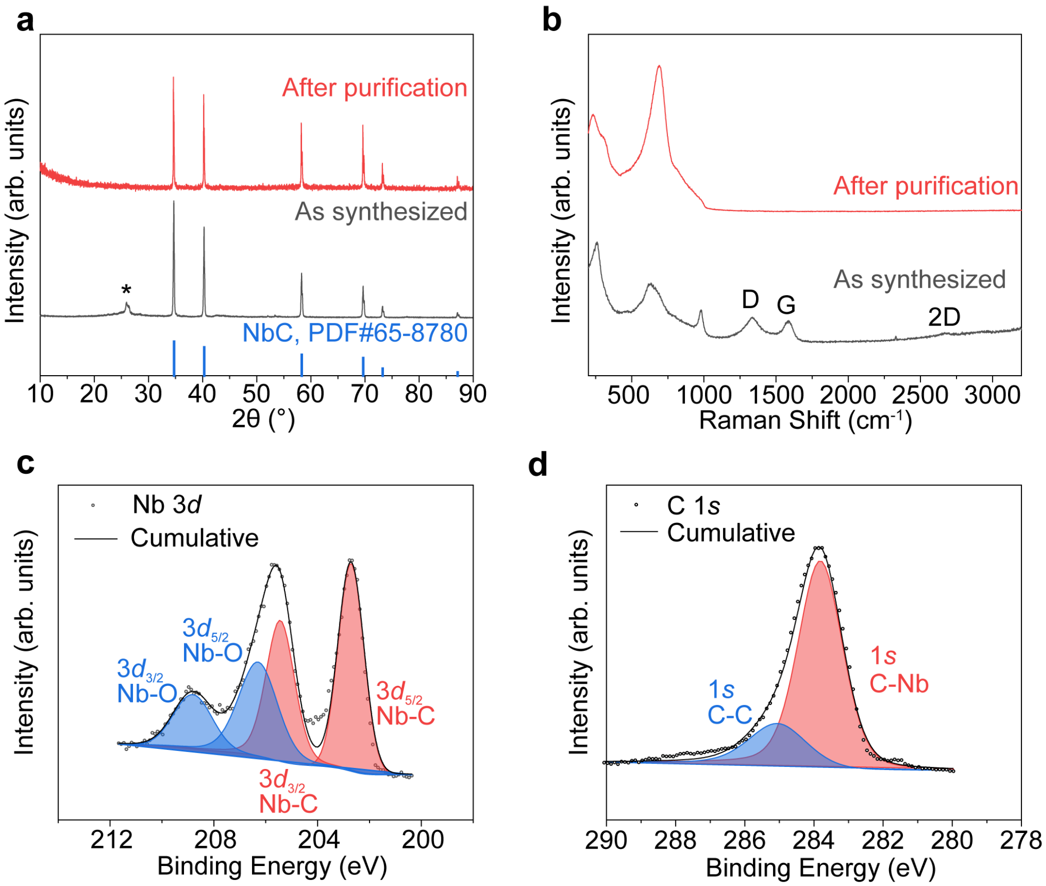

Supplementary Fig. 31. Purification of NbC nanocrystals. (**a**) X-ray diffraction (XRD) patterns of the as-synthesized mixture of NbC/graphene, and the NbC product after purification. The star denotes the peak from the graphene support. (**b**) Raman spectra of the as-synthesized mixture of NbC/graphene, and the NbC product after purification. (**c**) X-ray photoemission spectroscopy (XPS) fine spectrum of Nb in the purified NbC product. (**d**) XPS fine spectrum of C in the purified NbC product.

After purification, no graphene peak was identified in the XRD pattern, and no graphene band was detected in the Raman spectrum, demonstrating the efficient removal of graphene. The XPS confirms the chemical states of Nb and C in NbC. The Nb-O could be due to the surface oxidation, and the C-C peak could be from the absorbed carbon in air.

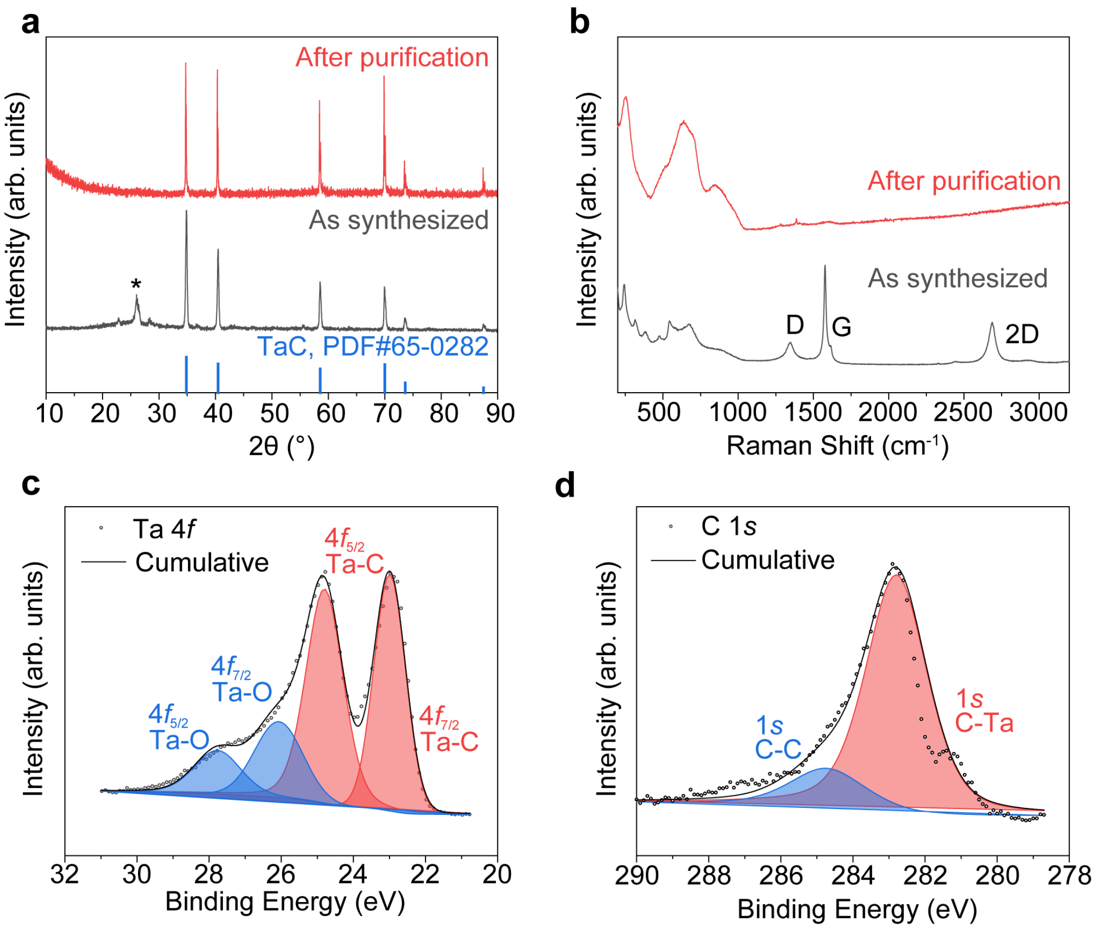

Supplementary Fig. 32. Purification of TaC nanocrystals. (**a**) X-ray diffraction (XRD) patterns of the as-synthesized mixture of TaC/graphene, and the TaC product after purification. The star denotes the peak from the graphene support. (**b**) Raman spectra of the as-synthesized mixture of TaC/graphene, and the TaC product after purification. (**c**) X-ray photoemission spectroscopy (XPS) fine spectrum of Ta in the purified TaC product. (**d**) XPS fine spectrum of C in the purified TaC product.

After purification, no graphene peak was identified in the XRD pattern, and no graphene band was detected in the Raman spectrum, demonstrating the efficient removal of graphene. The XPS confirms the chemical states of Ta and C in TaC. The Ta-O could be due to the surface oxidation, and the C-C peak could be from the absorbed carbon in air.

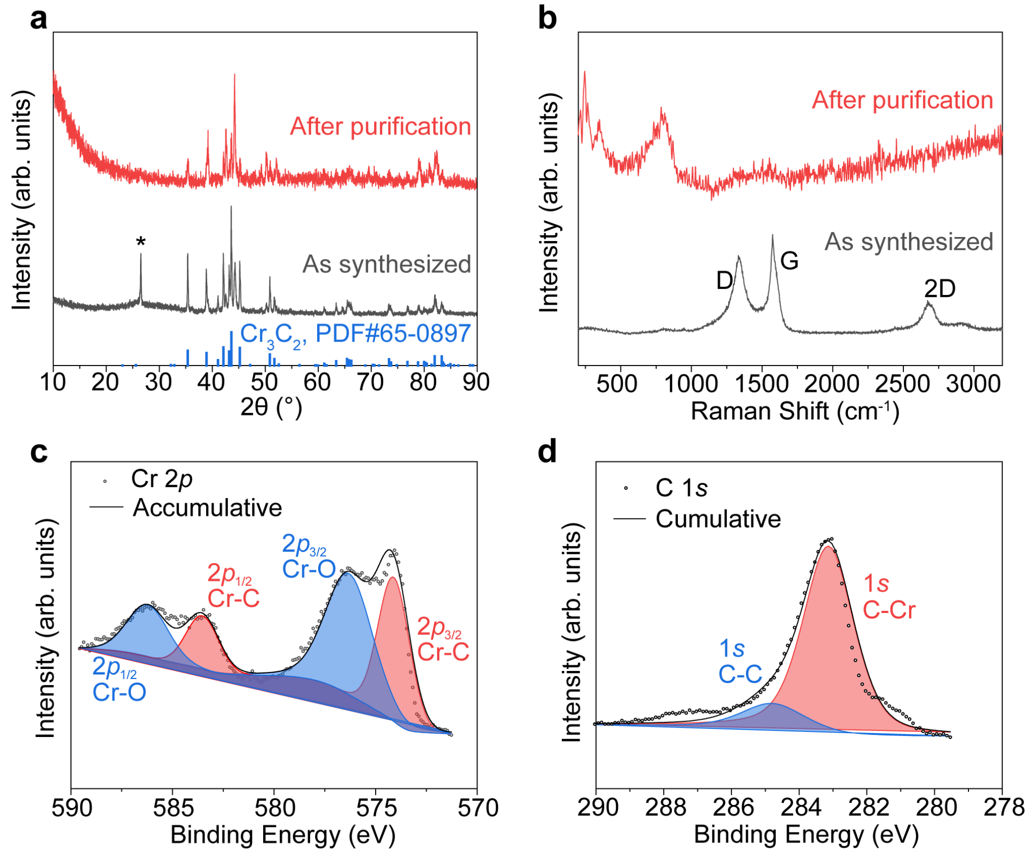

Supplementary Fig. 33. Purification of Cr_3C_2 nanocrystals. (**a**) X-ray diffraction (XRD) patterns of the as-synthesized mixture of Cr_3C_2/graphene, and the Cr_3C_2 product after purification. The star denotes the peak from the graphene support. (**b**) Raman spectra of the as-synthesized mixture of Cr_3C_2/graphene, and the Cr_3C_2 product after purification. (**c**) X-ray photoemission spectroscopy (XPS) fine spectrum of Ta of the purified Cr_3C_2 product. (**d**) XPS fine spectrum of C of the purified Cr_3C_2 product.

After purification, no graphene peak was identified in the XRD pattern, and no graphene band was observed in the Raman spectrum, demonstrating the efficient removal of graphene. The XPS confirms the chemical states of Cr and C in Cr_3C_2. The Cr-O could be due to the surface oxidation, and the C-C peak could be from the absorbed carbon in air.

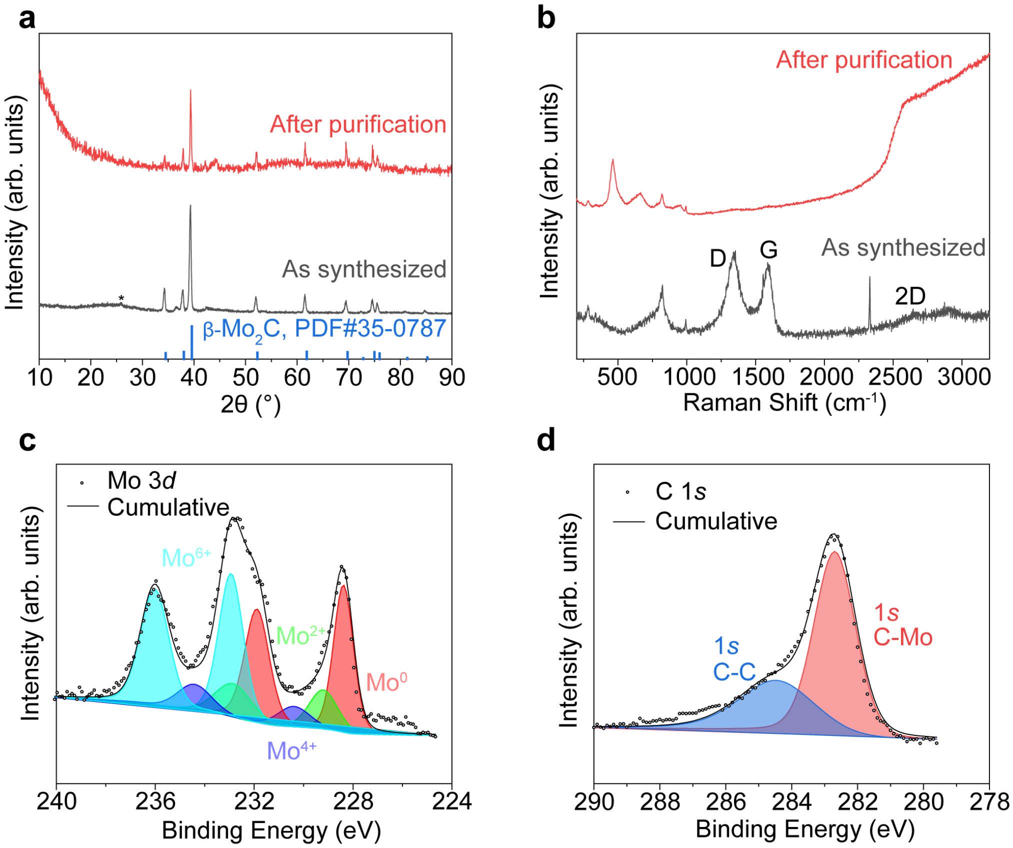

Supplementary Fig. 34. Purification of β-Mo_2C nanocrystals. (**a**) X-ray diffraction (XRD) patterns of the as-synthesized mixture of β-Mo_2C/graphene, and the β-Mo_2C product after purification. The star denotes the peaks from the graphene support. (**b**) Raman spectra of the as-synthesized mixture of β-Mo_2C/graphene, and the β-Mo_2C product after purification. (**c**) X-ray photoemission spectroscopy (XPS) fine spectrum of Ta in the purified β-Mo_2C product. (**d**) XPS fine spectrum of C in the purified β-Mo_2C product.

After purification, no graphene peak was identified in the XRD pattern, and no graphene band was observed in the Raman spectrum, demonstrating the efficient removal of graphene. The XPS confirms the chemical states of Mo and C in β-Mo_2C. The Mo-O could be due to the surface oxidation, and the C-C peak could be from the absorbed carbon in air.

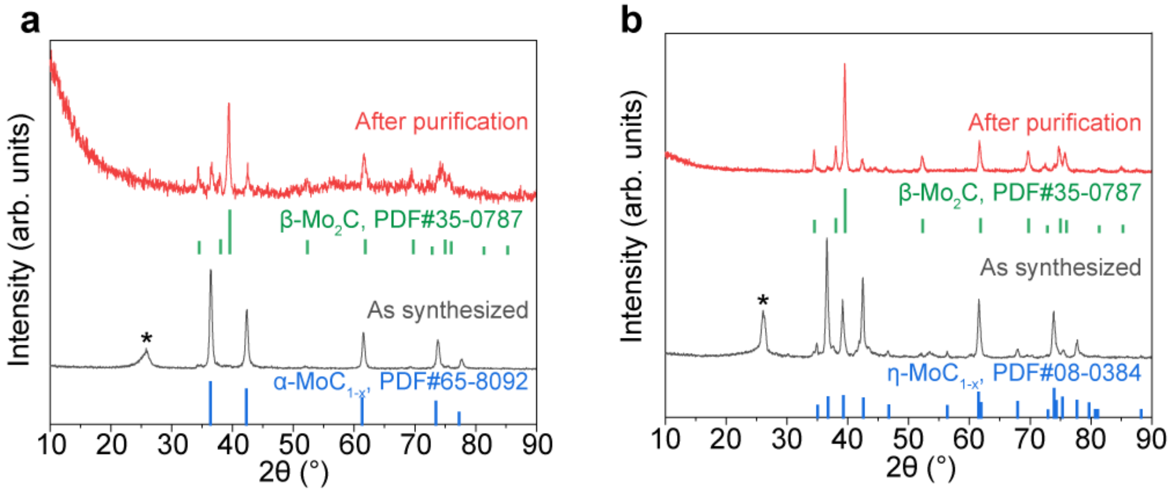

Supplementary Fig. 35. Purification of α-MoC_{1-x} and η-MoC_{1-x} nanocrystals. (**a**) X-ray diffraction (XRD) patterns of the as-synthesized mixture of α-MoC_{1-x}/graphene, and the product after purification. The star denotes the peak from the graphene support. (**b**) XRD patterns of the as-synthesized mixture of η-MoC_{1-x}/graphene, and the product after purification. The star (*) denotes the peak from graphene support.

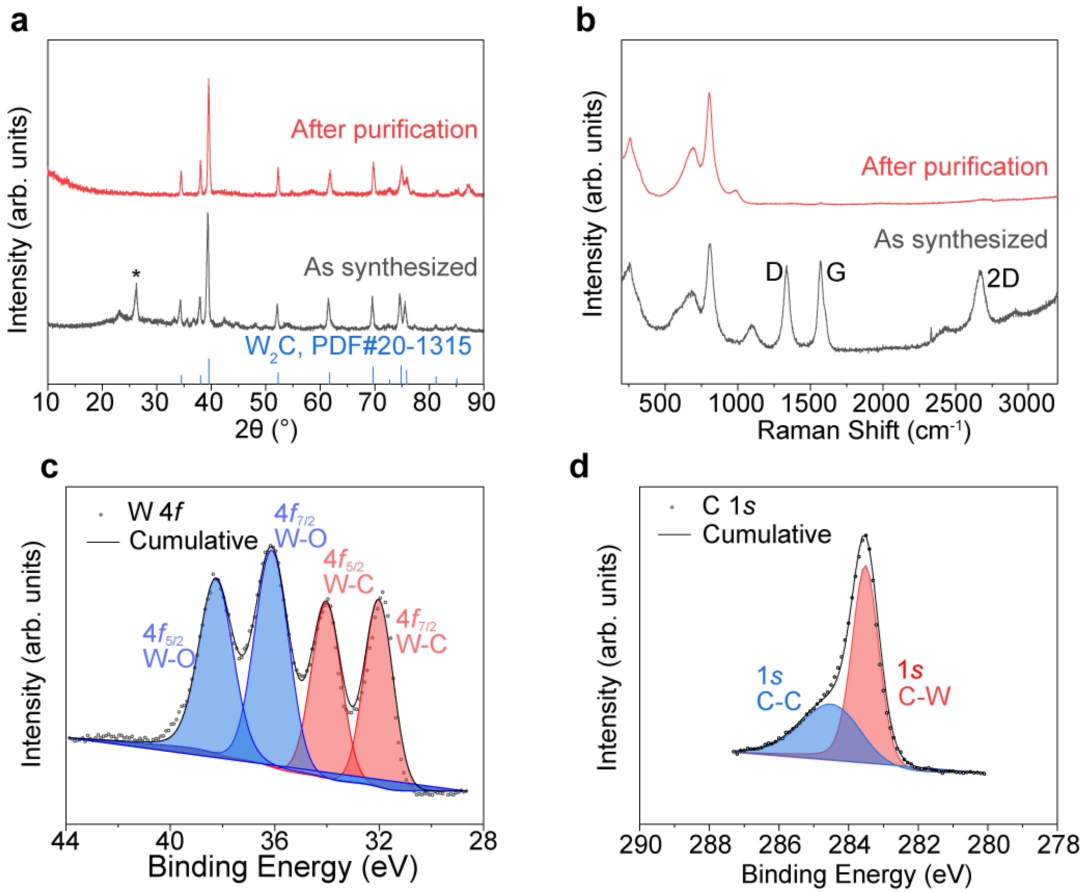

Supplementary Fig. 36. Purification of W_2C nanocrystals. (**a**) X-ray diffraction (XRD) patterns of the as-synthesized mixture of W_2C/graphene, and the product after purification. The star denotes the peak from the graphene support. (**b**) Raman spectra of the as-synthesized mixture of W_2C/graphene, and the W_2C product after purification. (**c**) X-ray photoemission spectroscopy (XPS) fine spectrum of W of the purified W_2C product. (**d**) XPS fine spectrum of C of the purified W_2C product.

After purification, no graphene peak was identified in the XRD pattern, and no graphene band was detected in the Raman spectrum, demonstrating the efficient removal of graphene. The XPS confirms the chemical states of W and C in W_2C. The W-O could be due to the surface oxidation, and the C-C peak could be from the absorbed carbon in air.

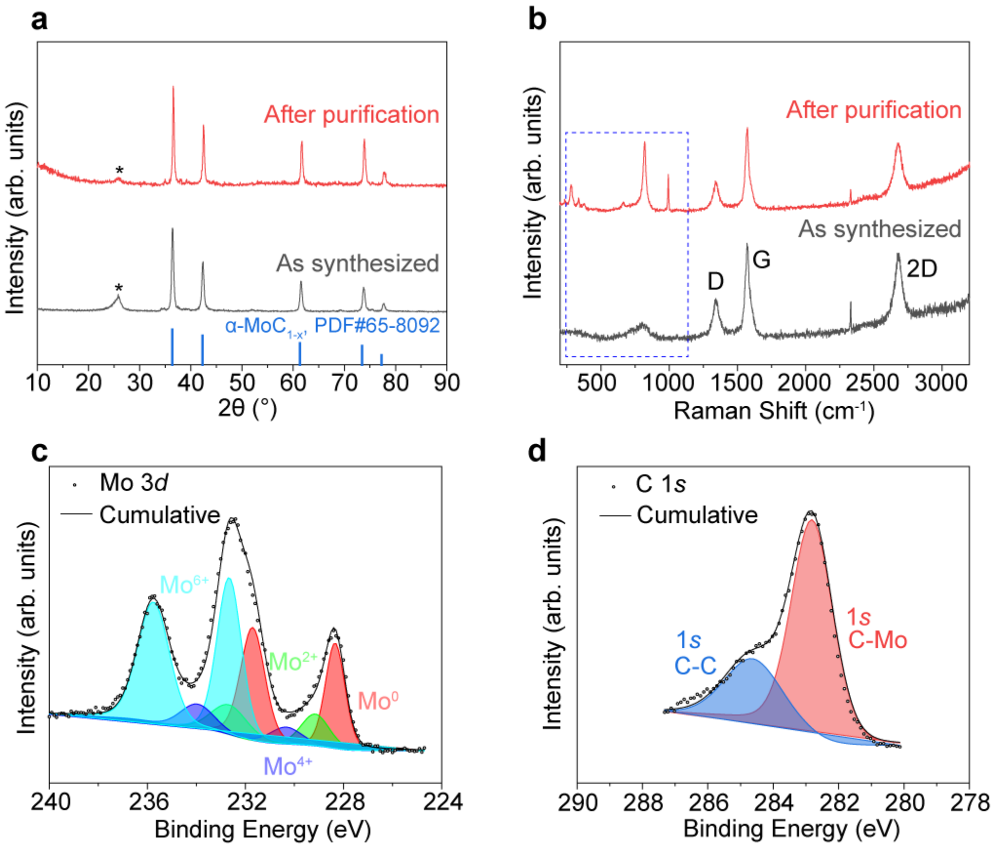

Supplementary Fig. 37. Purification of α-MoC_{1-x} nanocrystals. (**a**) X-ray diffraction (XRD) patterns of the as-synthesized mixture of α-MoC_{1-x}/graphene, and the product after purification. The star denotes the peak from the graphene support. (**b**) Raman spectra of the as-synthesized mixture of α-MoC_{1-x}/graphene, and the α-MoC_{1-x} product after purification. The blue rectangle indicates the Raman bands from carbide. (**c**) X-ray photoemission spectroscopy (XPS) fine spectrum of Mo in the purified α-MoC_{1-x} product. (**d**) XPS fine spectrum of C in the purified α-MoC_{1-x} product.

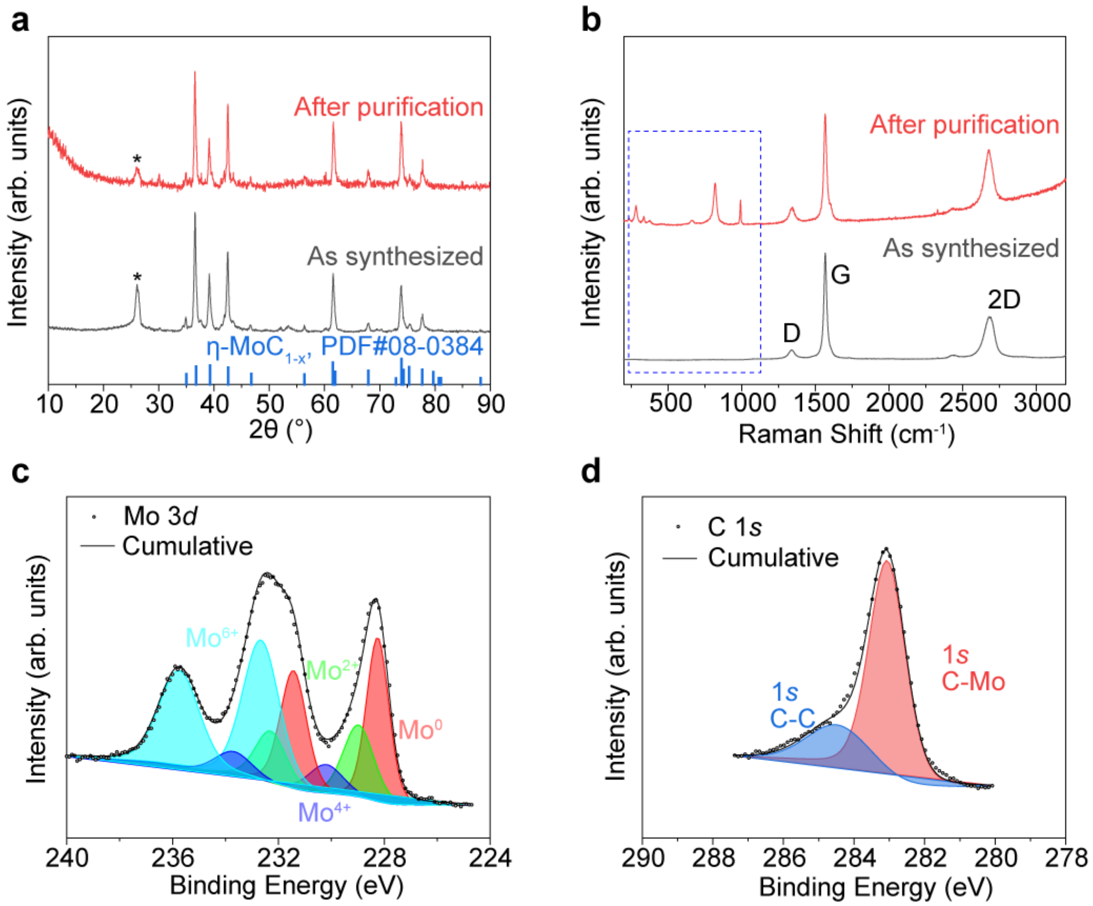

Supplementary Fig. 38. Purification of η-MoC_{1-x} nanocrystals. (**a**) XRD patterns of the as-synthesized mixture of η-MoC_{1-x}/graphene, and the product after purification. The star denotes the peak from the graphene support. (**b**) Raman spectra of the as-synthesized mixture of η-MoC_{1-x}/graphene, and the η-MoC_{1-x} product after purification. The dashed blue rectangle denotes the Raman bands from carbide. (**c**) X-ray photoemission spectroscopy (XPS) fine spectrum of Mo in the purified η-MoC_{1-x} product. (**d**) XPS fine spectrum of C in the purified η-MoC_{1-x} product.

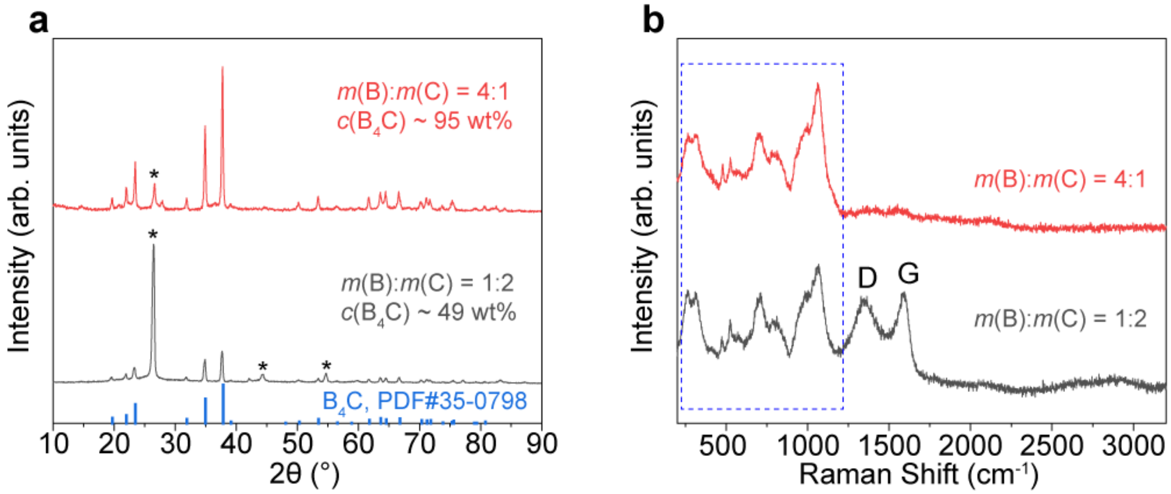

Supplementary Fig. 39. Improving the purity of B_4C. (**a**) X-ray diffraction (XRD) patterns of the B_4C product with the precursor mass ratios of *m*(B):*m*(C) = 1:2, and *m*(B):*m*(C) = 4:1. The star denotes the peak from the carbon support. (**b**) Raman spectra of the B_4C product with the precursor mass ratios of *m*(B):*m*(C) = 1:2, and *m*(B):*m*(C) = 4:1. The blue dashed rectangle denotes the Raman bands from B_4C.

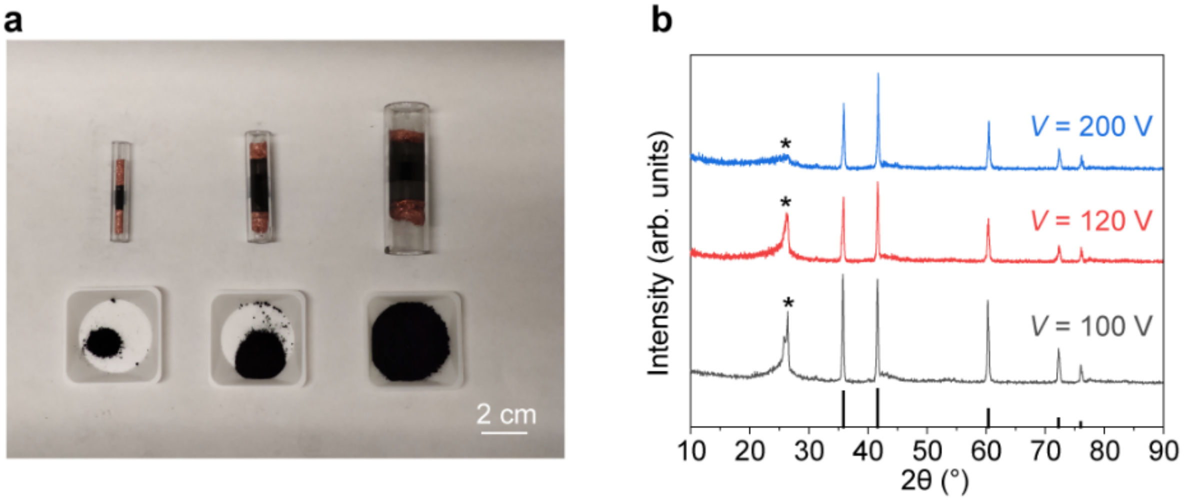

Supplementary Fig. 40. Scaling up of the flash Joule heating (FJH) process to gram scale. (**a**) Picture of the precursor (Ti and CB with the mass ratio of 1:1) in quartz tubes with different inner diameters of 0.4 cm, 0.8 cm, and 1.6 cm (top), and the corresponding product with final mass of 46 mg, 187 mg, and 0.96 g (bottom). (**b**) X-ray diffraction (XRD) patterns of the TiC products with the mass of ~50 mg, ~200 mg, ~1 g, under the FJH voltages (*V*) of 100 V, 120 V, and 200 V, respectively. The reference PDF card is TiC (#65-7994). The star (*) denotes the graphite (002) peak.

Supplementary Table 1. Parameters for simulation.

Parameters	Values
**Diameter**	4 mm
**Length**	20 mm
**Voltage**	80 ~ 120 V
**Electrical conductivity**	0.6 ~ 1.4 S m^{-1}
**Thermal conductivity**	0.1 ~ 10 W m^{-1} K^{-1}

Supplementary Table 2. Parameters for carbide synthesis

Precursors	Mass Ratios	Molar ratios	Mass (mg)	Resistance (Ω)	Voltage (V)	Time (ms)	Product	Crystal size (nm)
Ti:CB	1:1	1:4	50.0	2	120	500	TiC	30.7
$Zr(OH)_4$:CB	1:1	1:13	51.2	4	120	500	ZrC	40.6
HfO_2:CB	1:1	1:18	48.3	2	100	500	HfC	30.8
VO_2:CB	1:1	1:4	49.0	3	120	500	VC	26.6
$NbCl_5$:CB	1:1	1:23	50.3	2	120	500	NbC	24.1
$TaCl_5$:CB	1:1	1:30	50.2	2	120	500	TaC	28.7
Cr:CB	4:1	1:1	51.2	4	120	500	Cr_3C_2	14.6
$MoCl_3$:CB	1:1	1:17	50.3	2	30	1000	β-Mo_2C	22.3
$MoCl_3$:CB	1:1	1:17	48.6	1	60	1000	α-$MoC1_{1-x}$	17.6
$MoCl_3$:CB	1:1	1:17	49.7	1	120	500	η-MoC_{1-x}	17.4
WO_3:CB	1:1	1:19	48.7	2	100	1000	W_2C	25.1
B:CB	1:2	1:1	27.6	3	120	500	B_4C	--
SiO_2:CB	4:1	1:1.25	53.2	10	150	500	SiC	--

Supplementary References

1 Song, J. P., Tian, K. Y., Ma, L. X., Li, W. & Yao, S. C. The effect of carbon black morphology to the thermal conductivity of natural rubber composites. *Int. J. Heat. Mass. Tran.* **137**, 184-191 (2019).

2 Athanasopoulos, N., Koutsoukis, G., Vlachos, D. & Kostopoulos, V. Temperature uniformity analysis and development of open lightweight composite molds using carbon fibers as heating elements. *Compos. B. Eng.* **50**, 279-289 (2013).

3 Pak, A. Y. *et al.* Silicon carbide obtaining with DC arc-discharge plasma: synthesis, product characterization and purification. *Mater. Chem. Phys.* **271**, 124938-124944 (2021).

4 Lee, J. S., Oyama, S. T. & Boudart, M. Molybdenum carbide catalysts .1. Synthesis of unsupported powders. *J. Catal.* **106**, 125-133 (1987).

5 Xie, Z. *et al.* Preparation of nano-sized titanium carbide particles via a vacuum carbothermal reduction approach coupled with purification under hydrogen/argon mixed gas. *RSC Adv.* **7**, 9037-9044 (2017).

6 Dyjak, S., Norek, M., Polanski, M., Cudzilo, S. & Bystrzycki, J. A simple method of synthesis and surface purification of titanium carbide powder. *Int. J. Refract. Met. H* **38**, 87-91 (2013).

7 Luong, D. X. *et al.* Gram-scale bottom-up flash graphene synthesis. *Nature* **577**, 647-651 (2020).

8 Sathyanarayan Rao (2021). Blackbody Radiation Spectrum from Wien's Law and Planck's law (https://www.mathworks.com/matlabcentral/fileexchange/48253-blackbody-radiation-spectrum-from-wien-s-law-and-planck-s-law), MATLAB Central File Exchange. Retrieved April 26, 2021.

9 Wan, C., Regmi, Y. N. & Leonard, B. M. Multiple phases of molybdenum carbide as electrocatalysts for the hydrogen evolution reaction. *Angew. Chem. Int. Edit.* **53**, 6407-6410 (2014).

10 Ma, R. G. *et al.* Ultrafine molybdenum carbide nanoparticles composited with carbon as a highly active hydrogen-evolution electrocatalyst. *Angew. Chem. Int. Edit.* **54**, 14723-14727 (2015).

11 Reddy, K. M., Liu, P., Hirata, A., Fujita, T. & Chen, M. W. Atomic structure of amorphous shear bands in boron carbide. *Nat. Commun.* **4**, 2483 (2013).

12 Harima, H. Raman scattering characterization on SiC. *Microelectron. Eng.* **83**, 126-129 (2006).
